# Perceptions of physical activity and sedentary behaviour guidelines among end-users and stakeholders: a systematic review

**DOI:** 10.1186/s12966-022-01245-9

**Published:** 2022-03-02

**Authors:** Heather Hollman, John A. Updegraff, Isaac M. Lipkus, Ryan E. Rhodes

**Affiliations:** 1 Behavioural Medicine Laboratory, School of Exercise Science, Physical and Health Education, University of Victoria, PO Box 1700 STN CSC, Victoria, B.C. V8W 2Y2 Canada; 2 Kent State University, Kent, USA; 3 Duke University, Durham, USA

**Keywords:** Physical activity, Sedentary behavior, Review

## Abstract

**Background:**

Many of the world’s population, across all age groups and abilities, are not meeting or even aware of internationally recommended physical activity (PA) and sedentary behaviour (SB) guidelines. In order to enhance awareness and uptake, guidelines should be perceived positively by targeted users. The purpose of this study was to review the literature on end-user and stakeholder perceptions of PA and SB guidelines.

**Methods:**

The electronic databases APA PsycInfo, CINAHL, MEDLINE, and SPORTDiscus, using EBSCOhost Research Platform, and Web of Science were searched from inception to June, 2021 with keyword synonyms for “perceptions”, “PA guidelines”, and “SB guidelines”. Studies of any design that collected stakeholder and/or end-user responses to a PA and/or SB guideline were included and assessed for risk of bias. The PA and/or SB guideline could be any type of official form (e.g., national documents, organizational guidelines, expert consensus statements, etc.) from any country, that targets individuals at the regional, provincial/statewide, national, or international level, and includes all types of guidelines (e.g., strength, aerobic, clinical, nonclinical, screen-time, sitting, etc.). Data were extracted and analyzed using thematic synthesis.

**Results:**

After screening 1399 abstracts and applying citation screening, 304 full-texts were retrieved. A total of 31 articles met the inclusion criteria. End-users and stakeholders for PA guidelines across all age groups expressed the need for simplified language with more definitions, relatable examples and imagery, and quantification of PA behaviours. There was concern for the early years and child PA guidelines leading to guilt amongst parents and the SB guidelines, particularly the recommendations to limit screen-time, being unrealistic. General age group PA guidelines were not perceived as usable to populations with differing abilities, clinical conditions, and socioeconomic status. Guidelines that targeted clinical populations, such as persons with multiple sclerosis and persons with spinal cord injury, were well received.

**Conclusions:**

There is a clear need to balance the evidence base with the pragmatic needs of translation and uptake so that the guidelines are not ignored or act as a barrier to actual engagement.

**Supplementary Information:**

The online version contains supplementary material available at 10.1186/s12966-022-01245-9.

## Background

Regular physical activity (PA) can prevent and treat numerous noncommunicable diseases and improve mental health and quality of life of people of all ages [1, 2]. As a result, several national and international PA guidelines have been developed and implemented with the aim to provide stakeholders (i.e., policymakers, researchers, and healthcare providers) and end-users (i.e., target population, parents, early childhood educators) guidance in the minimal doses required for obtaining health benefits. Several countries, including Canada, USA, the UK, and Australia, released their first set of PA guidelines in the 1990s and each have undergone several modifications since. For example, the history of the American PA guidelines started in 1975 when the American College of Sports Medicine (ACSM) published the first specific exercise recommendations *Guidelines for Graded Exercise Testing and Exercise Prescription* [3]. In tandem, the ACSM also published exercise dose recommendations for improving and maintaining physical fitness in 1978 and then adjusted them in 1990, and 1998 with a focus more towards health outcomes [4].

The first internationally recommended PA guidelines were released in 2010 from the WHO as a result of its global mandate on promoting PA for public health and the limited existence of national guidelines on PA for public health in low- and middle- income countries [5]. These guidelines were updated in 2020 to include specific recommendations on PA for pregnant and postpartum women and people living with chronic conditions or disability as well as the addition of sedentary behaviour (SB) guidelines [6]. In response to accumulating evidence that SB contributes to health independently from PA [7, 8], SB-related messages have also been included in several national PA guidelines for adults and children in the UK, Australia, New Zealand, Canada, Germany, and Norway [9]. However, SB guidelines have been scrutinized for having an inconsistent evidence base [6, 9, 10]. There is no doubt that SBs, particularly screen-time (ST), have effects on health, but research is still preliminary to inform SB thresholds [6].

Despite these efforts in developing PA and SB guidelines, uptake is still concerningly low, as 31.1% of adults age 15 years or older, worldwide, remain physically inactive (i.e., not meeting one of the following: 1) 30 min of moderate-intensity PA on at least 5 days every week, 2) 20 min of vigorous-intensity PA on at least 3 days every week, or 3) an equivalent combination achieving 600 metabolic equivalent minutes per week) [11]. Among adolescents worldwide, 80.3% of 13–15-year-olds were not meeting 60 min of moderate-to-vigorous intensity PA per day and girls were less active than boys [11]. Over half of children in Canada, USA, and Australia are not meeting PA or ST recommendations put forward by their national governing bodies [12–14].

This discrepancy between available guidelines and PA participation may exist partly due to limited awareness of PA and/or SB guidelines [15–18]. However, information about acceptance and reception of guidelines may have even greater importance given that reception of guidelines has influence on attitudes, perception of capability, and intention to enact the guidelines [19, 20]. The persuasion-communication model proposes that in order for behaviour change to occur, message acceptance, reception, and retention are necessary [21]. Despite numerous PA and SB guideline reviews and epidemiology reports, no review has ever collected impressions of PA and/or SB guidelines.

The purpose of this study was to review the literature of end-user (i.e., targeted populations, parents, early childhood educators) and stakeholder (i.e., researchers, policymakers, healthcare practitioners) perceptions towards PA and SB guidelines put forward by national and international governing bodies. A specific aim was to separately evaluate the perceptions of PA and SB guidelines that targeted each age group (i.e., early years, children and youth, adults, older adults) and clinical population and evaluate the perceptions by end-user and stakeholder.

## Methods

This systematic review was registered in PROSPERO CRD42020207107 and follows the PRISMA items for reporting systematic reviews [22].

### Eligibility criteria

Inclusion criteria was any study design where perceptions of PA and/or SB guidelines by end-users and/or stakeholders were collected. Responses could be in the form of perceptions, attitudes, or opinions, and be from people of all ages and abilities. The PA and/or SB guideline could be any type of official form (e.g., national documents, organizational guidelines, expert consensus statements, etc.) from any country, that targets individuals at the regional, provincial/statewide, national, or international level, and includes all types of guidelines (e.g., strength, aerobic, clinical, nonclinical, ST, sitting, etc.). Studies were excluded if they evaluated perceptions of end-users and stakeholders to PA or SB in general instead of a specific PA or SB guideline (e.g. [6, 23, 24]), if only knowledge or awareness of the guidelines was collected, if they weren’t available in English language, and if they weren’t published in a peer-reviewed journal.

### Information sources, search strategy, and study selection

Two systematic searches, search #1 for PA guidelines and search #2 for SB guidelines, were conducted across APA PsycInfo, SPORTDiscus, MEDLINE, and CINAHL, using EBSCOhost Research Platform, and Web of Science. Search #1 included key word synonyms of “perception” and “PA guidelines” and search #2 included key word synonyms of “perception” and “SB guidelines”. See Supplementary Table 1 for full search strategies.

We managed study selection using Covidence software [25], a web-based screening and data extraction tool recommended by the Cochrane Collaboration. After identifying and removing duplicate records, one reviewer (HH) screened the citation information for each record using the set criteria. Covidence allows each reviewer to select “Yes”, “No”, or “Maybe” to include or exclude imported articles. If the reviewer thought “Maybe”, it was labeled as “Yes” and moved to the full-text screen. Full-text screening was completed by HH and RR. Disagreements between reviewers were resolved through discussion until consensus was reached. When consensus was not reached, the last author (RR) provided a final decision. Studies found to be ineligible during the full-text screening were recorded along with reasons for exclusion. Study selection for both searches was completed at the end of June, 2021.

### Data collection process and data items

Data extraction was completed by HH and a research assistant (see acknowledgement section) for search #1, HH alone for search #2, and both were reviewed by RR. Extracted information included authors, country of research, whether it was national or regional, the sample size and participant characteristics, study design, the specific PA and/or SB guideline assessed, the outcome variable (e.g., perception, attitude, or opinion of the PA and/or SB guideline), and the results of each study (see Supplementary Table 2).

### Risk of bias assessment

A risk of bias analysis was conducted across studies, applying the VAKS tool for studies with a qualitative design [26], the NIH Quality Assessment Tool for Observational Cohort and Cross-Sectional Studies for the studies that employed surveys [27], and the RoB 2 tool for the study with randomized controlled trial design [28]. The VAKS tool is divided into five subjects: formal requirements, credibility, transferability, dependability, and confirmability with all subjects weighed equally. Subjects have a range between five and seven criteria, of which each is scored on a four-point scale from 1 = “totally disagree” to 4 = “totally agree”. For each subject, the criterion scores are added and divided by the number of relevant criteria for each subject. The scores of the subjects are added to create a final score and if the result is 15 or above, the article is recommended, between 10 and 14, it is recommended with reservations, and below 10, it is not recommended [26]. The NIH Quality Assessment Tool for Observational Cohort and Cross-Sectional Studies includes 14 criteria scored “Yes”, “No”, or “Other (cannot determine, not applicable, or not reported)” and the overall quality rating (i.e. good, fair, or poor) is up to the discretion of the researcher with consideration of all the criteria and their potential for bias [27]. The RoB 2 assesses bias within domains of deviations from the randomization process, intended interventions, missing outcome data, measurement of the outcome, and selection of the reported results [28]. The methodological quality of studies were independently scored by HH and a research assistant for search #1 and HH for search #2. Discrepancies of search #1 were resolved through discussion and consensus between HH and the research assistant and the final scores for both searches were reviewed by RR.

### Analysis

Qualitative results were synthesized using thematic synthesis [29] where first the text was coded ‘line-by-line’ by HH using QSR NVivo Software [30]. Quantitative data were included in the synthesis to support qualitative findings. There was a pre-set organization of themes by PA, SB, type of PA (i.e., aerobic, strength) or SB (i.e., ST, sitting), age and/or clinical status that the guideline targeted. The first stage of analysis involved an iterative process of reading and re-reading studies to find codes that informed the primary research purpose of perceptions of end-users and stakeholders of PA and/or SB guidelines. Both raw data, such as participant quotes, as well as author interpretations were coded. Abstracts, results, and discussion sections were screened because findings of qualitative studies may not be limited to the results section [29]. As HH coded each study, codes were added, and new ones were developed when necessary. All studies were eventually coded using the final coding scheme (see Supplementary Table 3). Upon completion of coding, “descriptive themes” were developed based on the data retrieved from the primary studies, and then further refined into “analytical themes” generated by the interpretations of the authors of this study. Triangulation was performed by RR who reviewed the codes and themes and suggested revisions when indicated.

## Results

### Study selection

**Fig. 1:**
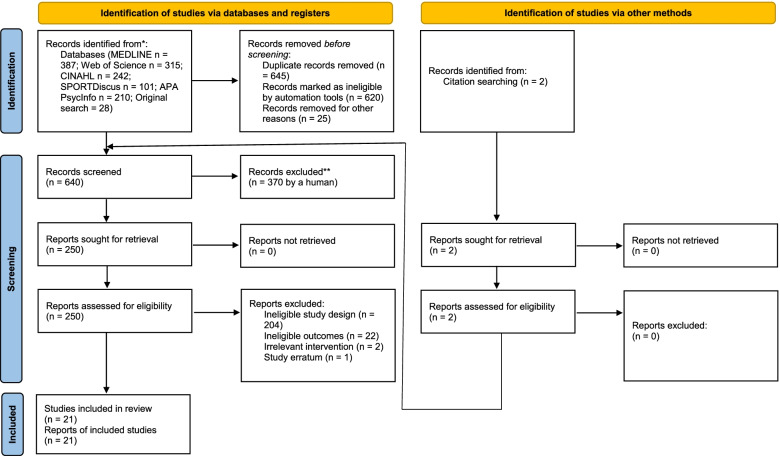
PRISMA Flow Diagram for Search #1

**Fig. 2:**
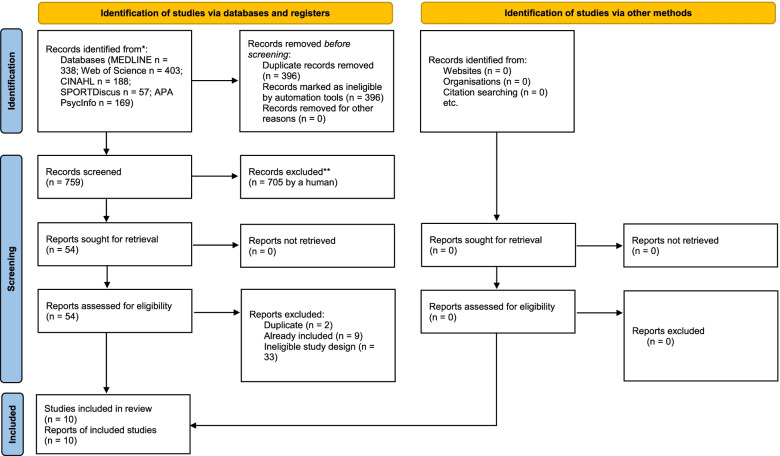
PRISMA Flow Diagram for Search #2

Thirty-one articles were selected for inclusion and the PRISMA Flow Diagram for search #1 and #2 can be seen in Figs. 1 and 2 respectively. For search #1 (see Fig. 1), after the removal of 645 duplicates, 640 records were screened by titles and abstracts, resulting in 370 records excluded. The remaining 250 records were screened full text and 228 studies were excluded because they a) employed ineligible study designs (*n* = 204), b) measured ineligible outcomes (*n* = 22), c) evaluated an irrelevant intervention (*n* = 2), and d) were a study erratum (*n* = 1). For search #2 (see Fig. 2), after removal of 396 duplicates, 759 records were screened by titles and abstracts, resulting in 705 records excluded. The remaining 54 records were screened full text and 44 studies were excluded because they a) were additional duplicates not identified by Covidence automation tool (*n* = 2), b) were already identified in search #2 (*n* = 9), and c) employed an ineligible study design (*n* = 33). A total of 21 studies from search #1 and 10 studies from search #2 with 31 independent samples passed the inclusion criteria and were included for analysis (Supplementary Table 2; Figs. 1 and 2).

### Study characteristics

Full data extraction details are provided in Supplementary Table 2 and study characteristics are presented in Table 1. Of the 31 selected articles, *n* = 15 targeted guidelines for early years (0–5 years), *n* = 11 for children and youth (6–17 years), *n* = 6 for adults (18–64 years), *n* = 4 for older adults (65 years and older), and *n* = 4 for specific clinical populations (i.e., cancer survivors, persons with multiple sclerosis (MS), and persons with spinal cord injury (SCI)). Six papers included perceptions of guidelines that targeted more than one population (e.g., children plus adults). Perceptions were collected from PA guidelines (*n* = 10), SB guidelines (*n* = 3), and ST guidelines (*n* = 5) alone, and PA and SB guidelines (*n* = 10), and PA and ST guidelines (*n* = 3) combined. Over 70% of the studies employed qualitative interviews (*n* = 15) and/or focus groups (*n* = 11). Characteristics of the PA, SB, and ST guidelines can be viewed in the Supplementary Table 2.

**Table 1:** Overall Study Characteristics

Characteristic	Number of Datasets	Percentages
Total studies/samples (*N* = 31) Number of unique data sets (*N* = 31)		
**Location**		
Canada	13	42%
USA	7	23%
Australia	6	19%
UK	3	10%
South Korea	1	3%
Sweden	1	3%
**Study design**		
Interviews	15	48%
Focus groups	11	35%
Cross-sectional surveys	10	32%
Workshops	1	3%
Randomized controlled trial	1	3%
Comments from news posts	1	3%
Electronic telephone interview	1	3%
**User**		
End-user	25	81%
Stakeholder	9	29%
Child & youth	5	16%
General adult	2	6%
Older adult	2	6%
Parent	21	68%
Researcher	3	10%
Policymaker	3	10%
Healthcare provider	8	26%
Public health practitioner	2	6%
Educator	3	10%
Recreation/sport practitioner	2	6%
Early childhood educator	2	6%
Early childhood educator trainee	1	3%
Undergraduate student	1	3%
Office worker	2	6%
Clinical population	6	19%
**Type of guideline**		
Physical activity	23	74%
Sedentary behaviour	13	42%
Screen**-**time	8	26%
**Guideline target**		
Early years (0–4)	15	48%
Child & youth (5–17)	11	35%
Adult (18–64)	6	19%
Older adult (65 +)	4	13%
Clinical population	4	13%
**User characteristic**		
African American	1	3%
Somali	1	3%
Low socioeconomic status	2	6%

Parents and early childhood educators were both classified as end-users

Risk of bias analyses for studies that employed qualitative designs (*n* = 26), seen in Table 2, demonstrated that most articles (*n* = 23) were recommended, two studies were recommended with reservation, and one article was not recommended. Risk of bias analyses for the cross-sectional survey studies (*n* = 9), seen in Table 3, demonstrated above 50% in five articles (56%) and below 50% in 4 articles (44%). The main limitations were lack of sample size justification, lack of measuring exposure of interest prior to measuring an outcome, lack of examining varying levels of exposure as related to the outcome, lack of assessing exposure more than once, lack of blinding outcome assessor to the exposure status of participants, and lack of measuring and adjusting for key potential confounding variables and their impact on the relationship between exposure(s) and outcome(s). The randomized controlled trial was deemed high risk (Table 4) because 24% of randomized participants dropped out and there was no evidence that the result wasn’t biased by the drop-outs.

**Table 2:** Risk of Bias Scores for the Qualitative Studies

Authors	Formal Requirements	Credibility	Transferability	Dependability	Confirmability	Total	Recommendation
Beck et al., 2016 [31]	3.00	4.00	3.40	4.00	2.50	16.90	R
Bentley et al., 2015 [32]	3.00	3.57	4.00	4.00	2.50	17.07	R
Berry et al., 2010 [33]	3.00	3.86	4.00	4.00	2.83	17.69	R
Bevington et al., 2020 [34]	2.50	2.71	3.40	2.67	1.17	12.98	RWR
Birken et al., 2015 [35]	3.00	4.00	3.20	4.00	3.00	17.20	R
Brown & Smolenaers, 2018 [36]	2.50	4.00	3.00	4.00	2.67	16.20	R
Carson et al., 2014 [37]	3.00	4.00	3.40	3.33	3.00	17.40	R
Evans et al., 2011 [38]	2.50	3.86	3.20	4.00	3.00	16.60	R
Faulkner et al., 2016 [39]	3.00	3.86	3.40	4.00	2.83	17.09	R
Gardner et al., 2017 [40]	3.00	4.00	3.40	4.00	3.83	18.23	R
Golden et al., 2020 [41]	3.00	3.60	3.40	4.00	3.00	17.00	R
Hale et al., 2019 [42]	2.70	4.00	4.00	4.00	3.30	18.00	R
Handler et al., 2019 [43]	3.00	4.00	3.40	4.00	3.50	17.90	R
Hattersley et al., 2009 [44]	3.00	3.57	3.40	4.00	2.33	16.30	R
Hinkley & McCann, 2018 [45]	3.00	3.29	3.20	3.83	2.50	15.82	R
Huxtable et al., 2018 [46]	3.33	4.00	4.00	4.00	2.83	18.17	R
Irwin et al., 2005 [47]	2.67	4.00	3.40	4.00	2.67	16.74	R
Learmonth et al., 2019 [48]	3.00	4.00	4.00	4.00	3.50	18.50	R
Martin Ginis et al., 2018 [49]	2.50	4.00	3.60	4.00	3.20	17.30	R
Neher et al., 2020 [50]	3.00	3.86	4.00	4.00	3.67	18.53	R
Nobles et al., 2020 [51]	2.83	4.00	3.40	4.00	3.50	17.73	R
Riazi et al., 2017 [52]	3.00	4.00	3.40	4.00	3.17	17.57	R
Sebastiao et al., 2015 [53]	3.00	4.00	3.40	4.00	3.50	17.90	R
Slater et al., 2010 [54]	2.50	2.71	3.20	2.80	2.33	13.505	RWR
Stanley et al., 2020 [55]	3.00	4.00	3.40	4.00	3.00	17.40	R
The Health Perspective, 2002 [56]	1.33	1.00	1.60	1.83	1.00	6.76	NR

*R* Recommended, *RWR* Recommended with Reservations *NR* Not Recommended

**Table 3:** Risk of Bias Scores for the Quantitative Surveys

	1	2	3	4	5	6	7	8	9	10	11	12	13	14	Total	Percentage
Birken et al., 2015 [35]	+	+	NR	+	-	+	+	-	+	-	+	-	NA	+	8/12	67%
Carson et al., 2013 [57]	+	+	-	+	-	+	+	-	+	-	+	-	NA	+	8/13	62%
Faught et al., 2020 [58]	+	+	-	-	-	-	+	-	-	-	+	-	-	-	4/14	29%
Jarvis et al., 2021 [59]	+	+	NA	+	+	-	+	-	+	-	+	-	NA	+	8/12	67%
Learmonth et al., 2019 [48]	+	+	+	+	-	-	+	-	-	-	+	-	NA	-	6/13	46%
Martin Ginis et al., 2018 [49]	+	+	NR	+	-	-	+	NA	NA	-	+	-	NA	-	5/10	50%
Park et al., 2015 [60]	+	+	-	+	-	-	+	NA	NA	-	+	-	NA	-	5/11	45%
Sebastiao et al., 2015 [53]	+	+	NR	+	-	+	NR	-	+	+	+	-	NA	-	7/11	64%
Slater et al., 2010 [54]	+	+	+	+	-	-	+	-	+	-	+	+	NA	-	8/13	62%

+  = Yes;—= No, *NA* Not available, *NR* Not recorded

1. Was the research question or objective in this paper clearly stated?

2. Was the study population clearly specified and defined?

3. Was the participation rate of eligible persons at least 50%?

4. Were all the subjects selected or recruited from the same or similar populations (including the same time period)? Were inclusion and exclusion criteria for being in the study prespecified and applied uniformly to all participants?

5. Was a sample size justification, power description, or variance and effect estimates provided?

6. For the analyses in this paper, were the exposure(s) of interest measured prior to the outcome(s) being measured?

7. Was the timeframe sufficient so that one could reasonably expect to see an association between exposure and outcome if it existed?

8. For exposures that can vary in amount or level, did the study examine different levels of the exposure as related to the outcome (e.g., categories of exposure, or exposure measured as continuous variable)?

9. Were the exposure measures (independent variables) clearly defined, valid, reliable, and implemented consistently across all study participants?

10. Was the exposure(s) assessed more than once over time?

11. Were the outcome measures (dependent variables) clearly defined, valid, reliable, and implemented consistently across all study participants?

12. Were the outcome assessors blinded to the exposure status of participants?

13. Was loss to follow-up after baseline 20% or less?

14. Were key potential confounding variables measured and adjusted statistically for their impact on the relationship between exposure(s) and outcome(s)?

**Table 4:** Risk of Bias Score for the Randomized Controlled Trial

	Randomization Process	Intended Interventions	Adhering to Intervention	Missing Outcome Data	Measurement of Outcome	Reported Result	Risk-of-Bias Judgment
Tennant et al., 2019 [61]	SC	LR	LR	HR	LR	LR	HR

*SC* some concerns, *LR* low risk of bias, *HR* high risk of bias

Representative quotes for the themes of perceptions of guidelines that targeted each age group and clinical condition can be seen in Supplementary Table 4. The results will be presented divided by age groups.

### Early years (0–4 years)

The PA, SB, and ST guidelines were homogenous (Supplementary Table 2) with the exception of an older PA guideline which recommended 30–90 min of PA per day [47], the Maternal Child and Health PA recommendations of active play every day without a time threshold [46], and the recent 24-Hour Movement Guidelines recommending up to 1 h of ST per day for children 1–2 years [52, 55]. Of the 15 articles, 13 collected responses of original guideline documents, one from a health professional communication document [46], and one from a guidelines draft [52].

#### Overall positive support for PA and SB/ST guidelines

End-users (parents) and stakeholders (pediatricians) thought the PA guidelines were achievable and realistic [32, 46, 54, 57]. It was often reported that children of this age were “naturally active” and already exceeded PA guidelines [32, 46, 47, 52]. End-users (parents) thought the SB guidelines were acceptable, sensible, clear, and understandable [32, 35, 37] and stakeholders (pediatricians) agreed or strongly agreed (96%) with the SB guidelines [57].

#### Meeting the SB/ST guidelines is unrealistic

The principles of the SB/ST guidelines were commended [35, 45] however the majority of end-users reported that meeting the SB/ST guidelines was unrealistic [31, 35–37, 41, 42, 55] and only half (51%) of pediatricians thought they were very realistic [57]. Parents often relied on the benefits of SB/ST such as its use as a “babysitter” to occupy children while parents complete household chores, using the stroller to transport children to where they can be active, improving family communication, regulating behaviour, and using it for educational programming [32, 35–37, 41, 42].

#### Guidelines should be tailored to individuals and encourage achievable goals rather than rigid times

End-users liked the simplicity and clarity of the guidelines [37, 42, 52, 55] although many thought that the PA guidelines were too general (i.e., one-size-fits-all) [32, 34, 42]. Both end-users and stakeholders reported that the guidelines should be more tailored to developmental stage, physical ability, [32, 36, 52, 55] and socioeconomic status [42, 55]. End-users preferred when guidelines were broken down into more achievable steps that could be built upon [32, 34–36, 42, 52, 55].

#### Guidelines should provide suggestions and visuals of examples that stakeholders can apply

Both end-users and stakeholders reported that PA and SB/ST guidelines could result in guilt to parents [32, 37, 42, 52, 55]. Suggestions included providing examples and more details around quantifying PA and SB/ST behaviours [32, 34, 36, 37, 42, 46, 54, 55]. There was some confusion over “what counted” as ST [36] and parents wanted specific strategies to help them achieve the recommended ST and PA behaviours [37, 38, 46, 54, 55]. End-users wanted more of a focus on images rather than text that were reflective of diverse activities and diverse individuals that were colourful and inviting [34, 36, 42, 52, 55].

### Children & youth (5–17 years)

The PA, SB, and ST guidelines were homogenous (Supplementary Table 2). Of the 11 articles, 9 collected responses of original guideline documents, one from an end-user guide [59], and one from a guidelines draft [39].

#### Conflicting positive and negative perception of the guidelines

Stakeholders (pediatricians) and end-users (parents) generally responded positively (i.e., agreeable, message believability, realistic) to the concept of PA and SB/ST guidelines [38, 54, 57, 59], however youth themselves weren’t as agreeable [38, 39]. Youth expressed anger to the idea of limiting television time [38] or a disengaged attitude because their future health wasn’t an immediate concern [39]. End-users (parents and youth) often reported that the ST guidelines were unrealistic [38, 44, 54] with 20% fewer pediatricians considering the PA and SB guidelines very realistic compared to the early years [57]. Users reported guidelines that integrated PA and SB/ST behaviours provided a holistic approach and didn’t affect message processing (i.e., degree to which guideline information is thought about and predictive of attitudes and behaviours) [39, 43, 61].

#### End-users need more guidance on monitoring and integrating PA and SB guideline recommendations into their daily lives

Parents reported difficulty quantifying PA and SB behaviours when children and youth spent varying times in multiple environments (home, school, day care, community centre) every day [39]. In order to be achievable, the guidelines should include examples of what different levels of PA intensities look like and how they can be incorporated into daily life, taking into account the many barriers such as time, energy, and costs [34, 38, 39, 43, 51, 54]. End-users (parents and youth) did not like the language of the PA guidelines, describing it as academic, clinical, and inaccessible [51], nor the one-size-fits-all recommendations of the PA and SB guidelines [34]. They preferred when guideline messages broke recommendations down into more achievable goals [34, 39, 51].

#### Guidelines should be tailored to ability and developmental stage

End-users reported that the guidelines should include more examples and imagery of children with varying levels of ability, mobility, pain, gender, and age [34, 38, 39, 43, 51, 54]. Parents of children and youth with disabilities requested more relatable examples and language as the 24-Hour Movement Guidelines were not inclusive nor compatible with the abilities and needs of children and youth with disabilities [43].

### Adults (18–64 years)

The PA, SB, and ST guidelines were homogenous with the exception of two older versions of PA guidelines recommending 60 min of PA per day [33, 56]. The SB guideline from Gardner and colleagues [40] was unique to desk-workers (Supplementary Table 2). Of the six articles, four collected responses from original guideline documents, one from an end-user guide [33], and one from a guidelines draft [58].

#### The PA and SB guidelines are too simplistic

End-users and stakeholders primarily reported negative feedback regarding the PA guidelines [33, 34, 51, 56, 58] and SB guidelines [40, 58]. End-users felt that the guidelines were too simplistic [33] and along with health practitioners, thought they didn’t account for varying abilities, health situations, and socioeconomic status’ of individuals [51, 58]. The simplicity resulted in end-users questioning the credibility of the PA and SB guidelines [33, 40]. In order to enhance confidence in the guidelines, end-users requested evidence of the reasoning behind the guidelines as well as the associated health and social benefits [34, 40, 51].

#### Guidelines need to be more understandable to varying literacy levels with focus on strength-based language

There were common concerns of the literacy level of the PA and SB guidelines with end-users reporting that certain terms, such as “sedentary”, “vigorous”, “intensity”, “movement”, and “muscle-strengthening” needed to be either removed, modified, or described [51, 58]. End-users and stakeholders preferred the message of increasing PA to eventually meet PA and SB guidelines, because evidence exists demonstrating activity levels below such levels will still result in health benefits [34, 51, 56, 58]. End-users desired messages that motivated, encouraged, and invited people to work towards meeting the guidelines [51, 58]. End-users also wanted to see more examples of activities of varying intensities that people with varying abilities, cultural backgrounds, and ages could easily fit into their routines [33, 34, 40, 51, 58].

### Older Adults (65 + years)

The PA guidelines were homogenous with the exception of an older PA guideline that recommended 60 min of PA per day [33] ( Supplementary Table 2). Of the four articles, two collected responses of original guideline documents, two from end-user guides [33, 53], and one from a guidelines draft [58].

#### PA guideline content needs to apply more understandable and inclusive language

End-users reported overall negative perceptions of the content and layout of the PA guidelines [33, 51, 53]. Difficult language and technical terminology, such as “aerobic”, “intensity”, “moderate”, and “muscle-strengthening” resulted in confusion by many end-users [51, 53].

#### Guidelines should be tailored to varying abilities and cultural backgrounds

End-users wanted images of PA that were representative and realistic for them (e.g. chair-based activity) [33, 51, 53]. Further, end-users wanted clear instructions on how to achieve varying levels of activity [58] along with applicable health benefits, such as changes in mobility [51]. In contrast to the original guidelines, end-users responded positively to public health brochures that included more detailed instructions and visual illustrations [53].

### Clinical population guidelines

Two PA guidelines targeted cancer survivors, recommending either 150 min of moderate- to vigorous-intensity PA or 150 min of moderate-intensity or 75 min of vigorous intensity PA per week [50, 60]. One PA guideline targeted persons with MS, recommending 30 min of aerobic exercise 2 × per week and resistance exercise training 2 × per week [48]. One PA guideline targeted persons with SCI which included two specific guidelines, one for cardiorespiratory fitness the second for cardiometabolic health benefits [49] (Supplementary Table 2). Of the four articles, three collected responses of original guideline documents and one from a clinical implementation document [50].

#### Guidelines are unclear for cancer survivors

Two articles collected perceptions of cancer health professionals to PA guidelines [50, 60]. Health practitioners thought PA guidelines were important but also unclear and were surprised that they were not unique to cancer patients [50, 60].

#### Overall positive support for PA guidelines that target persons with MS and persons with SCI

Perceptions of end-users and stakeholders to PA guidelines specific for persons with SCI [49] and persons with MS [48] were overall positive. Persons with MS reported that the PA guidelines were acceptable and appropriate [48] while health practitioners and end-users reported overall confidence in the PA guidelines for persons with SCI, except for the cardiometabolic health guideline for people with tetraplegia [49]. There were a few concerns including confusion that could result from not knowing which of the two guidelines for persons with SCI to implement (fitness or health), that the term “physical activity” may be more appropriate than “exercise”, and that the importance of improving fitness may be overshadowed by the cardiometabolic health guideline [49].

## Discussion

The purpose of this review was to evaluate end-user and stakeholder perceptions of PA and SB guidelines, sub-divided by age group and clinical population. This is the first review of its kind, providing valuable information to researchers and policymakers about the overall reception of each guideline by end-users and stakeholders. The systematic search retrieved 31 articles, of which the majority employed qualitative designs. Several negative responses were identified that, if addressed, may inform implementation strategies needed to ultimately improve uptake of guidelines [62].

There were many positive responses from both end-users and stakeholders to PA guidelines, particularly those that targeted early years and specific clinical populations (i.e., persons with MS and persons with SCI). Most users felt that toddlers and preschoolers were naturally active and could easily achieve the PA recommendations. Although there was overall consensus for the importance of PA and SB guidelines across age groups, there were several difficulties in understanding and applying them. The SB and ST guidelines for the early years were often perceived as unrealistic with parents relying on occupying their children with ST to accomplish housework and SB like strollers to transport children to places that they can be active. Parents of older children did not rely on ST as much; however, children and youth reported anger and frustration towards ST guidelines and did not see immediate value in following PA guidelines. Adults and older adults reported that the PA and SB guidelines were too simplistic and requested evidence in support of the guidelines.

PA and SB guidelines are typically written with the evidence-base in mind over the usefulness to end-users, however, it is important that when guidelines are communicated to end-users, that the information is comprehendible and obtainable [63]. The following are several recommendations that researchers and policymakers could consider to improve perception and ultimately uptake of PA and SB guidelines. Across the age-group guidelines, users requested more lay language, definitions of terms, and descriptions of behaviour quantification. For example, terms such as “moderate- and vigorous-intensity” may be described with reference to the Talk Test that states individuals should be able to speak short sentences during moderate-intensity PA but only a few words at a time during vigorous-intensity PA [64]. Users requested examples that people of varying ages, abilities, and socioeconomic and cultural backgrounds could employ to achieve guideline recommendations. These examples should be demonstrated in visually appealing ways with clear instructions when guidelines are being communicated to end-users.

Users, particularly parents, need strategies for implementing ST rules, for example through increased self-efficacy of minimizing ST [65]. Parents also need to be informed that school-based PA is inadequate for resulting in ongoing PA behaviours into adulthood [66, 67]. Parents requested strategies for promoting child PA during evenings and weekends, while faced with the barriers of limited time and busy family schedules. Family-based PA interventions have shown promise for PA levels of both children and parents and should be tailored to the psychosocial environment of the family [68, 69]. Youths requested more information of reachable health-benefits and consequences of movement behaviours, for example, improved cognitive outcomes including math, science, and reading achievements or ST resulting in increased obesity, sleep problems, depression, and anxiety [70, 71]. Further, youth may choose reasons other than those resulting in health or cognitive benefits for being active. For example, research has shown autonomous motivation (e.g., intrinsic motivation, integrated motivation, and identified regulation) as an important predictor for leisure-time PA in children and adolescents [72, 73]. Therefore, guideline communication efforts targeting youth could highlight the importance of choosing PA that is interesting, enjoyable, and important to the individual [73]. Adults and older adults requested more information about evidence behind the guidelines which could be easily translated from the numerous systematic reviews that have been conducted and published by global experts in PA guideline development.

Health practitioners and clinical populations prefer PA guidelines that target clinical populations. In response to feedback from mothers of children and youth with disabilities, authors from Handler and colleagues (2019) developed an Ability Toolkit to supplement the children & youth PA guidelines [74]. This toolkit provides examples of movement behaviours that are more applicable to children and youth with disabilities. End-user perceptions of this toolkit have not yet been collected but responses could help inform additional tailored toolkits for other target populations.

PA and SB/ST guidelines are put forward to achieve maximal health benefits. That said, our findings revealed that stakeholders and end-users were not always receptive to one-size-fits-all threshold messages and that either adding to or replacing these messages with phrases that encourage overall more movement or less SB/ST may be better received. More research is needed to determine whether PA interventions that recommend changes in movement behaviours phrased in more general terms is still effective for resulting in health benefits.

### Limitations

Despite strengths of this review, there are limitations worth mentioning. The review was limited by the search terms, search engines employed, and peer-reviewed articles in English. The literature was limited by insufficient consistency of outcome measures across the quantitative data that prevented any form of quantitative analysis and an inadequate amount of articles to break down perceptions of each guideline by specific end-user or stakeholder. Articles that collected perceptions of recently developed PA guidelines for target or clinical populations, such as pregnant and postpartum women [75, 76] or the 2018 American College of Sports Medicine Roundtable Exercise Guidelines for Cancer Survivors [77], were not available.

## Conclusions

Guidelines have been evolving for several decades now with minimal awareness and levels of enactment across the globe. Available end-user and stakeholder perceptions indicate modifications, such as more lay language, definitions, and implementation strategies, are needed when guidelines are being communicated to end-users. Perhaps a set of behavioural guidelines that target specific end-users and stakeholders for each guideline may be the most appropriate step forward in actualizing behaviours and habits.

## Supplementary Information

**Additional file 1:** **Supplementary Table 1. **Full Search Strategies for Each Database.

**Additional file 2:** **Supplementary Table 2.** Extraction Data of End-user and Stakeholder Perceptions of Physical Activity (PA) or Sedentary Behaviour (SB) Guidelines.

**Additional file 3:** **Supplementary Table 3.** Codes for the Final Coding Scheme.

**Additional file 4:** **Supplementary Table 4.** Selected Quotes for Each Theme.

## Data Availability

The datasets used and/or analysed during the current study are available from the corresponding author on reasonable request.

## Abbreviations

PA: Physical activity
SB: Sedentary behaviour
ST: Screen-time
PRISMA: Preferred Reporting Items for Systematic Reviews and Meta-Analyses
MS: Multiple sclerosis
SCI: Spinal cord injury

## References

[1] Kraus WE, Powell KE, Haskell WL, Janz KF, Campbell WW, Jakicic JM (2019). Physical activity, all-cause and cardiovascular mortality, and cardiovascular disease. Med Sci Sports Exerc.

[2] Poitras VJ, Gray CE, Borghese MM, Carson V, Chaput JP, Janssen I, et al. Systematic review of the relationships between objectively measured physical activity and health indicators in school-aged children and youth. Appl Physiol Nutr Metab. 2016;41(6):S197–239. 10.1139/apnm-2015-0663 27306431

[3] American College of Sports Medicine (1975). Guidelines for graded exercise testing and exercise prescription.

[4] Blair SN, LaMonte MJ, Nichaman MZ (2004). The evolution of physical activity recommendations: how much is enough?. Am J Clin Nutr.

[5] World Health Organization. Global recommendations on physical activity for health. Recomm Mond Sur Act Phys Pour Santé. 2010;58. 26180873

[6] Bull FC, Al-Ansari SS, Biddle S, Borodulin K, Buman MP, Cardon G (2020). World Health Organization 2020 guidelines on physical activity and sedentary behaviour. Br J Sports Med.

[7] Biswas A, Oh PI, Faulkner GE, Bajaj RR, Silver MA, Mitchell MS (2015). Sedentary time and Its association with risk for disease incidence, mortality, and hospitalization in adults. Ann Intern Med.

[8] Mahmood S, MacInnis RJ, English DR, Karahalios A, Lynch BM (2017). Domain-specific physical activity and sedentary behaviour in relation to colon and rectal cancer risk: a systematic review and meta-analysis. Int J Epidemiol.

[9] Stamatakis E, Ekelund U, Ding D, Hamer M, Bauman AE, Lee I-M (2019). Is the time right for quantitative public health guidelines on sitting? A narrative review of sedentary behaviour research paradigms and findings. Br J Sports Med.

[10] Dempsey PC, Biddle SJH, Buman MP, Chastin S, Ekelund U, Friedenreich CM (2020). New global guidelines on sedentary behaviour and health for adults: broadening the behavioural targets. Int J Behav Nutr Phys Act.

[11] Hallal PC, Andersen LB, Bull FC, Guthold R, Haskell W, Ekelund U (2012). Global physical activity levels: surveillance progress, pitfalls, and prospects. The Lancet.

[12] Friel CP, Duran AT, Shechter A, Diaz KMUS (2020). children meeting physical activity, screen time, and sleep guidelines. Am J Prev Med.

[13] Hinkley T, Salmon J, Okely AD, Crawford D, Hesketh K (2012). Preschoolers’ physical activity, screen time, and compliance with recommendations. Med Sci Sports Exerc.

[14] PujadasBotey A, Bayrampour H, Carson V, Vinturache A, Tough S. Adherence to Canadian physical activity and sedentary behaviour guidelines among children 2 to 13 years of age. Prev Med Rep. 2015;3(3):14–20. 10.1016/j.pmedr.2015.11.012 PMC4733064 26844180

[15] Dale LP, LeBlanc AG, Orr K, Berry T, Deshpande S, Latimer-Cheung AE (2016). Canadian physical activity guidelines for adults: are Canadians aware?. Appl Physiol Nutr Metab Physiol Appl Nutr Metab.

[16] Knox ECL, Taylor IM, Biddle SJH, Sherar LB (2015). Awareness of moderate-to-vigorous physical activity: can information on guidelines prevent overestimation?. BMC Public Health.

[17] LeBlanc AG, Boyer C, Borghese MM, Chaput J-P, Leduc G, Tremblay MS (2016). canadian physical activity and screen time guidelines: do children know?. Health Behav Policy Rev.

[18] Piercy KL, Bevington F, Vaux-Bjerke A, Hilfiker SW, Arayasirikul S, Barnett EY (2020). Understanding contemplators’ knowledge and awareness of the physical activity guidelines. J Phys Act Health.

[19] Rebar A, Rhodes RE, Tenenbaum G, Ecklund R (2020). Progression of motivation models in exercise science: where we have been and where we are heading. Handbook of sport psychology.

[20] Rhodes RE, McEwan D, Rebar AL (2019). Theories of physical activity behaviour change: a history and synthesis of approaches. Psychol Sport Exerc.

[21] McGuire W, Lindzey G, Aronson E (1985). Attitudes and Attitude Change. In The Handbook of Social Psychology.

[22] Page MJ, McKenzie JE, Bossuyt PM, Boutron I, Hoffmann TC, Mulrow CD (2021). The PRISMA 2020 statement: an updated guideline for reporting systematic reviews. BMJ.

[23] Piercy KL, Troiano RP, Ballard RM, Carlson SA, Fulton JE, Galuska DA (2018). The physical activity guidelines for Americans. JAMA.

[24] Ross R, Chaput J-P, Giangregorio LM, Janssen I, Saunders TJ, Kho ME (2020). Canadian 24-hour movement guidelines for adults aged 18–64 years and adults aged 65 years or older: an integration of physical activity, sedentary behaviour, and sleep. Appl Physiol Nutr Metab.

[25] Veritas Health Innovation. Covidence systematic review software [Internet]. Melbourne, Australia; Available from: www.covidence.org.

[26] Schou L, Høstrup H, Lyngsø EE, Larsen S, Poulsen I. Validation of a new assessment tool for qualitative research articles. J Adv Nurs. 2012;68(9):2086–94. 10.1111/j.1365-2648.2011.05898.x 22168459

[27] National Heart, Lung, and Blood Institute. Quality Assessment Tool for Observational Cohort and Cross-Sectional Studies [Internet]. Available from: https://www.nhlbi.nih.gov/health-topics/study-quality-assessment-tools

[28] Sterne JAC, Savović J, Page MJ, Elbers RG, Blencowe NS, Boutron I (2019). RoB 2: a revised tool for assessing risk of bias in randomised trials. BMJ.

[29] Thomas J, Harden A (2008). Methods for the thematic synthesis of qualitative research in systematic reviews. BMC Med Res Methodol.

[30] QSR International Pty Ltd. NVivo [Internet]. 2020. https://www.qsrinternational.com/nvivo-qualitative-data-analysis-software/home

[31] Beck AL, Takayama J, Badiner N, Halpern-Felsher B (2015). Latino parents’ beliefs about television viewing by infants and toddlers. J Health Care Poor Underserved.

[32] Bentley GF, Jago R, Turner KM (2015). Mothers’ perceptions of the UK Physical Activity and Sedentary Behaviour Guidelines for the Early Years (Start Active, Stay Active): A qualitative study. BMJ OPEN..

[33] Berry TR, Witcher C, Holt NL, Plotnikoff RC (2010). A qualitative examination of perceptions of physical activity guidelines and preferences for format. Health Promot Pract.

[34] Bevington F, Piercy KL, Olscamp K, Hilfiker SW, Fisher DG, Barnett EY (2020). The move your way campaign: encouraging contemplators and families to meet the recommendations from the physical activity guidelines for Americans. J Phys Act Health.

[35] Birken CS, Lichtblau B, Lenton-Brym T, Tucker P, Maguire JL, Parkin PC (2015). Parents’ perception of stroller use in young children: a qualitative study. BMC Public Health.

[36] Brown A, Smolenaers E (2018). Parents’ interpretations of screen time recommendations for children younger than 2 years. J Fam Issues.

[37] Carson V, Clark M, Berry T, Holt NL, Latimer-Cheung AE (2014). A qualitative examination of the perceptions of parents on the Canadian sedentary behaviour guidelines for the early years. Int J Behav Nutr Phys Act.

[38] Evans CA, Jordan AB, Horner J (2011). Only two hours?: A qualitative study of the challenges parents perceive in restricting child television time. J Fam Issues.

[39] Faulkner G, White L, Riazi N, Latimer-Cheung AE, Tremblay MS (2016). Canadian 24-Hour Movement Guidelines for Children and Youth: Exploring the perceptions of stakeholders regarding their acceptability, barriers to uptake, and dissemination. Appl Physiol Nutr Metab.

[40] Gardner B, Smith L, Mansfield L (2017). How did the public respond to the 2015 expert consensus public health guidance statement on workplace sedentary behaviour?. BMC Public Health.

[41] Golden SL, Blake JWC, Giuliano KK (2020). Parental decision-making: infant engagement with smartphones. Infant Behav Dev.

[42] Hale I, Amed S, Keidar S, Purcell M, Lee D, Farhadi D (2019). Parents’ perceptions of obesity prevention during infancy: a qualitative study. Can Med Assoc Open Access J.

[43] Handler L, Tennant EM, Faulkner G, Latimer-Cheung AE (2019). Perceptions of Inclusivity: the Canadian 24-hour movement guidelines for children and youth. Adapt Phys Act Q.

[44] Hattersley LA, Shrewsbury VA, King LA, Howlett SA, Hardy LL, Baur LA (2009). Adolescent-parent interactions and attitudes around screen time and sugary drink consumption: a qualitative study. Int J Behav Nutr Phys Act.

[45] Hinkley T, McCann JR (2018). Mothers’ and father’s perceptions of the risks and benefits of screen time and physical activity during early childhood: a qualitative study. BMC Public Health.

[46] Huxtable A, Millar L, Love P, Bell C, Whelan J (2018). Parental translation into practice of healthy eating and active play messages and the impact on childhood obesity: A mixed methods study. Nutrients..

[47] Irwin JD, He M, Bouck LMS, Tucker P, Pollett GL (2005). Preschoolers’ physical activity behaviours: parents’ perspectives. Can J Public Health Rev Can Sante Publique.

[48] Learmonth YC, Kinnett-Hopkins D, Motl RW (2019). Capitalising on the opinions of persons with multiple sclerosis to inform the main trial – participant opinions from participation in a feasibility study, a qualitative extension study. Disabil Rehabil.

[49] Martin Ginis KA, van der Scheer JW, Latimer-Cheung AE, Barrow A, Bourne C, Carruthers P (2018). Evidence-based scientific exercise guidelines for adults with spinal cord injury: an update and a new guideline. Spinal Cord.

[50] Neher M, LandénLudvigsson M, Enblom A. Preparedness to implement physical activity and rehabilitation Guidelines in routine primary care cancer rehabilitation: Focus group interviews exploring rehabilitation professionals’ perceptions. J Cancer Educ. 2021;36(4):779–86. 10.1007/s13187-020-01704-6 10.1007/s13187-020-01704-6 PMC8328890 32062799

[51] Nobles J, Thomas C, Banks Gross Z, Hamilton M, Trinder-Widdess Z, Speed C (2020). “Let’s talk about physical activity”: understanding the preferences of under-served communities when messaging physical activity guidelines to the public. Int J Environ Res Public Health.

[52] Riazi N, Ramanathan S, O’Neill M, Tremblay MS, Faulkner G (2017). Canadian 24-hour movement guidelines for the early years (0–4 years): exploring the perceptions of stakeholders and end users regarding their acceptability, barriers to uptake, and dissemination. BMC Public Health.

[53] Sebastiao E, Chodzko-Zajko W, Schwingel A (2015). The need to modify physical activity messages to better speak to older African American women: a pilot study. BMC Public Health.

[54] Slater A, Bowen J, Corsini N, Gardner C, Golley R, Noakes M (2010). Understanding parent concerns about children’s diet, activity and weight status: an important step towards effective obesity prevention interventions. Public Health Nutr.

[55] Stanley R, Jones R, Swann C, Christian H, Sherring J, Shilton T (2020). Exploring stakeholders’ perceptions of the acceptability, usability, and dissemination of the Australian 24-Hour movement guidelines for the early years. J Phys Act Health.

[56] The Health Perspective (2002). New exercise guidelines trigger concerns. Act Living.

[57] Carson V, LeBlanc CM, Moreau E, Tremblay MS (2013). Paediatricians’ awareness of, agreement with and use of the new Canadian physical activity and sedentary behaviour guidelines for children and youth zero to 17 years of age. Paediatr Child Health.

[58] Faught E, Walters AJ, Latimer-Cheung AE, Faulkner G, Jones R, Duggan M (2020). Optimal messaging of the Canadian 24-hour movement guidelines for adults aged 18–64 years and adults aged 65 years and older. Appl Physiol Nutr Metab.

[59] Jarvis JW, Berry TR, Carson V, Rhodes RE, Lithopoulos A, Latimer-Cheung AE (2021). Examining differences in parents’ perceptions of children’s physical activity versus screen time guidelines and behaviours. J Paediatr Child Health..

[60] Park J-H, Oh M, Yoon YJ, Lee CW, Jones LW, Kim SI (2015). Characteristics of attitude and recommendation of oncologists toward exercise in South Korea: a cross sectional survey study. BMC Cancer.

[61] Tennant EM, Tremblay MS, Faulkner G, Gainforth HL, Latimer-Cheung AE (2019). Exploring parents? message receipt and message enactment of the world’s first integrated movement behaviour guidelines for children and youth. J Health Commun.

[62] Tomasone JR, Kauffeldt KD, Morgan TL, Magor KW, Latimer-Cheung AE, Faulkner G (2020). Dissemination and implementation of national physical activity, sedentary behaviour, and/or sleep guidelines among community-dwelling adults aged 18 years and older: a systematic scoping review and suggestions for future reporting and research. Appl Physiol Nutr Metab.

[63] Latimer AE, Brawley LR, Bassett RL (2010). A systematic review of three approaches for constructing physical activity messages: What messages work and what improvements are needed?. Int J Behav Nutr Phys Act.

[64] Persinger R, Foster C, Gibson M, Fater DCW, Porcari JP (2004). Consistency of the talk test for exercise prescription. Med Sci Sports Exerc.

[65] Downing KL, Hinkley T, Salmon J, Hnatiuk JA, Hesketh KD (2017). Do the correlates of screen time and sedentary time differ in preschool children?. BMC Public Health.

[66] Cale L, Harris J (2006). School-based physical activity interventions: effectiveness, trends, issues, implications and recommendations for practice. Sport Educ Soc.

[67] Meyer U, Schindler C, Zahner L, Ernst D, Hebestreit H, van Mechelen W (2014). Long-Term Effect of a School-Based Physical Activity Program (KISS) on Fitness and Adiposity in Children: A Cluster-Randomized Controlled Trial. PLoS One.

[68] Brown HE, Atkin AJ, Panter J, Wong G, Chinapaw MJM, van Sluijs EMF (2016). Family-based interventions to increase physical activity in children: a systematic review, meta-analysis and realist synthesis. Obes Rev.

[69] Rhodes RE, Blanchard CM, Quinlan A, Naylor P-J, Warburton DER (2019). Family physical activity planning and child physical activity outcomes: A randomized trial. Am J Prev Med.

[70] Domingues-Montanari S (2017). Clinical and psychological effects of excessive screen time on children. J Paediatr Child Health.

[71] Fedewa A, Ahn S. The Effects of Physical Activity and Physical Fitness on Children’s Achievement and Cognitive Outcomes: a Meta-Analysis. Res Q Exerc Sport. 2011;82(3):521–35. 10.1080/02701367.2011.10599785 21957711

[72] Owen KB, Smith J, Lubans DR, Ng JYY, Lonsdale C (2014). Self-determined motivation and physical activity in children and adolescents: A systematic review and meta-analysis. Prev Med.

[73] Deci EL, Ryan RM. Intrinsic Motivation and Self-Determination in Human Behavior. New York: Plenum; 1985.

[74] Handler L, Tennant E, D’Urzo K, Latimer-Cheung AE. The Ability Toolkit. Available from: https://csepguidelines.ca/wp-content/uploads/2018/12/PA-New-Abilities-Toolkit-Final-ENG.pdf. Cited 2021 Sep 6.

[75] Lee R, Thain S, Tan LK, Teo T, Tan KH (2021). Asia-Pacific consensus on physical activity and exercise in pregnancy and the postpartum period. BMJ Open Sport Exerc Med.

[76] Mottola MF, Davenport MH, Ruchat S-M, Davies GA, Poitras VJ, Gray CE (2018). 2019 Canadian guideline for physical activity throughout pregnancy. Br J Sports Med.

[77] Campbell KL, Winters-Stone KM, Wiskemann J, May AM, Schwartz AL, Courneya KS (2019). Exercise guidelines for cancer survivors: consensus statement from international multidisciplinary roundtable. Med Sci Sports Exerc.

