# Supplementary material for: Perceptions of physical activity and sedentary behaviour guidelines among end-users and stakeholders: a systematic review
Source: Int J Behav Nutr Phys Act. 2022 Mar 2;19:21. doi: 10.1186/s12966-022-01245-9 (PMC8889734; doi:10.1186/s12966-022-01245-9)
Supplement: Supplementary file 1 — Additional file 1: Supplementary Table 1. Full Search Strategies for Each Database. [file 12966_2022_1245_MOESM1_ESM.docx]

**Supplementary Table 1**

*Full Search Strategies for Each Database*

| Search #1 | APA PsycInfo:  (perception* OR attitude* OR opinion*) AND  (“physical activity guideline” OR “physical activity guidelines” OR “exercise guideline” OR “exercise guidelines” OR “movement guideline” OR “movement guidelines” OR “fitness guideline” OR “fitness guidelines” OR “physical activity recommendation” OR “physical activity recommendations” OR “exercise recommendation” OR “exercise recommendations” OR “movement recommendation” OR “movement recommendations” OR “fitness recommendation” OR “fitness recommendations”)  CINAHL:  (perception* OR attitude* OR opinion*) AND  (“physical activity guideline” OR “physical activity guidelines” OR “exercise guideline” OR “exercise guidelines” OR “movement guideline” OR “movement guidelines” OR “fitness guideline” OR “fitness guidelines” OR “physical activity recommendation” OR “physical activity recommendations” OR “exercise recommendation” OR “exercise recommendations” OR “movement recommendation” OR “movement recommendations” OR “fitness recommendation” OR “fitness recommendations”) |
| --- | --- |
|  |  |
|  | MEDLINE:  (perception* OR attitude* OR opinion*) AND  (“physical activity guideline” OR “physical activity guidelines” OR “exercise guideline” OR “exercise guidelines” OR “movement guideline” OR “movement guidelines” OR “fitness guideline” OR “fitness guidelines” OR “physical activity recommendation” OR “physical activity recommendations” OR “exercise recommendation” OR “exercise recommendations” OR “movement recommendation” OR “movement recommendations” OR “fitness recommendation” OR “fitness recommendations”) |
|  | SPORTDiscus:  (perception* OR attitude* OR opinion*) AND  (“physical activity guideline” OR “physical activity guidelines” OR “exercise guideline” OR “exercise guidelines” OR “movement guideline” OR “movement guidelines” OR “fitness guideline” OR “fitness guidelines” OR “physical activity recommendation” OR “physical activity recommendations” OR “exercise recommendation” OR “exercise recommendations” OR “movement recommendation” OR “movement recommendations” OR “fitness recommendation” OR “fitness recommendations”) |
|  | Web of Science:  (perception* OR attitude* OR opinion*) AND  (“physical activity guideline” OR “physical activity guidelines” OR “exercise guideline” OR “exercise guidelines” OR “movement guideline” OR “movement guidelines” OR “fitness guideline” OR “fitness guidelines” OR “physical activity recommendation” OR “physical activity recommendations” OR “exercise recommendation” OR “exercise recommendations” OR “movement recommendation” OR “movement recommendations” OR “fitness recommendation” OR “fitness recommendations”) |
| Search #2 | APA PsycInfo:  (perception* or attitude* or opinion*) AND ("sedentary behaviour" or "sedentary behavior" or "screen-time" or "screen time" or inactivity or sitting) AND (guideline or guidelines or recommendation or recommendations) |
|  | CINAHL:  (perception* or attitude* or opinion*) AND ("sedentary behaviour" or "sedentary behavior" or "screen-time" or "screen time" or inactivity or sitting) AND (guideline or guidelines or recommendation or recommendations) |
|  | MEDLINE:  (perception* or attitude* or opinion*) AND ("sedentary behaviour" or "sedentary behavior" or "screen-time" or "screen time" or inactivity or sitting) AND (guideline or guidelines or recommendation or recommendations) |
|  | SPORTDiscus:  (perception* or attitude* or opinion*) AND ("sedentary behaviour" or "sedentary behavior" or "screen-time" or "screen time" or inactivity or sitting) AND (guideline or guidelines or recommendation or recommendations) |
|  | Web of Science:  (perception* or attitude* or opinion*) AND ("sedentary behaviour" or "sedentary behavior" or "screen-time" or "screen time" or inactivity or sitting) AND (guideline or guidelines or recommendation or recommendations) |
