# Supplementary material for: Perceptions of physical activity and sedentary behaviour guidelines among end-users and stakeholders: a systematic review
Source: Int J Behav Nutr Phys Act. 2022 Mar 2;19:21. doi: 10.1186/s12966-022-01245-9 (PMC8889734; doi:10.1186/s12966-022-01245-9)
Supplement: Supplementary file 2 — Additional file 2: Supplementary Table 2. Extraction Data of End-user and Stakeholder Perceptions of Physical Activity (PA) or Sedentary Behaviour (SB) Guidelines. [file 12966_2022_1245_MOESM2_ESM.docx]

**Supplementary Table 2**

*Extraction Data of End-user and Stakeholder Perceptions of Physical Activity (PA) or Sedentary Behaviour (SB) Guidelines*

| **Author** | **Country (National or Regional)** | **Sample** | **Study Design** | **Targeted PA, SB, or ST guideline** | **Outcome Variable (e.g., perception, attitude, opinion, satisfaction)** | **Perception Outcome Results** | | **Quality Score^1,2,3^** |
| --- | --- | --- | --- | --- | --- | --- | --- | --- |
| Beck et al., 2016 | USA (Regional) | **End-users:**  *n* = 26 (25 Latino mothers and 1 father with a child 6- to 36-months) | Qualitative Interviews (22 with only mothers, 1 with a father, and 3 with both parents) | **ST Guidelines (Early Years):**  American Academy Pediatrics ST guidelines for children recommending children <2 should refrain from watching television. | Participants were asked what they thought of the AAP guidelines on ST for children and how easier or difficult they would be to follow.  Interviews were transcribed verbatim and analyzed using a general inductive approach to generate themes. | **ST Guidelines (Early Years):**  Most participants reported that they would try to follow the recommendations and had confidence in replacing ST with other activities. They would be more likely to follow the guidelines if they were provided a rationale for the guidelines. Specific barriers to the guideline implementation were limiting ST when someone else was taking care of their child as well as screens used by other family members who shared a small living space. | | R |
| Bentley et al., 2015 | UK (Regional) | **End-users:**  *n* = 24 mothers of preschool children of varying socioeconomic (SES) status in both rural and urban communities  (5 low-SES, 6 mid-SES, 7 high-SES; 6 rural) | Qualitative Interviews | **PA and SB Guidelines (Early Years):**  PA guidelines for children <5 recommend  180 min of PA per day of any intensity spread throughout the day. PA mostly comprises of active play. SB guidelines recommend to minimize extended periods of SB (i.e., car seat, highchair, screen-viewing, crafts, reading, and puzzles). | Semi-structured interviews were conducted where participants were first asked about their family and child’s personality, the child’s routine (including PA and SB), and their views on their child’s PA and SB behaviours. They were then provided with the UK PA and SD guidelines for early years and asked to discuss their reactions to them.  Interviews were recorded, transcribed verbatim, and analyzed using an approach based on framework analysis.  Themes and quotes were provided. | **PA and SB Guidelines (Early Years):**  Initial reactions of mothers were that 180min seemed like a lot to achieve. However, once it was explained that the 180min could be broken up throughout the day with varying intensities, mother's felt that the guidelines were more achievable. Mothers found it difficult to differentiate between PA and sedentary time for activities involving play like dressing up and crafts. They also felt that television viewing didn't necessarily classify as sedentary time because their children were often moving while watching TV. Given the sporadic periods of activity for their children, mothers found it difficult to be able to discern whether the activity added up to the recommendations. | | R |
| Berry et al., 2010 | Canada (National) | **End-users:**  *n* = 22; five focus groups (5 female undergraduate students, 6 female office workers, 5 male office workers, 2 participants in a Type II diabetes rehabilitation program- 1 male, 1 female, and 4 males in an all-male cardiovascular rehabilitation group) | Qualitative Focus Groups, questionnaire for brief demographics (age and gender) and the Godin Leisure-Time Exercise Questionnaire | **PA Guidelines (Adults, Older Adults):**  Canada's PA guides aimed at adults (aged 18-55 years), older adults (older than 55 years) recommend 30-60 minutes of PA per day in periods of $\geq$10 minutes each, 4-7 days of endurance activities, 4-7 days of flexibility activities (daily for older adults), and 2-4 days a week of strength and balance activities. The older adult version provides examples of activities modified for older adults (e.g., under strength training the adult version suggests heavy yard wok and push-ups whereas the older adult guide recommends wall push-ups and lifting weights or soup cans). The guides have images of cartoons but the one for older adults has images of real people. | In the focus groups, participants were initially asked introductory questions regarding their motives for activity and how they changed over time. Next, they were asked, “Are you aware of Canada’s PA Guide?”, “Does Canada’s PA Guide attract you and what might make it more effective or attractive? Can you comment on how CPAG is presented?”, and “What do you want/need to see in terms of PA promotion materials in general? What would you prefer in terms of style and content?”  Focus groups were recorded, transcribed verbatim, and analyzed using cross-case analysis. Themes and quotes were provided. | **PA Guidelines (Adults, Older Adults):**  None of the participants were aware of the guidelines and when presented with them, participants criticized the style and content of the guidelines. They disliked the use of cartoons which led them to questioning the credibility of the guidelines. They also didn’t like the use of the rainbow to represent diverse types of PA. | R | |
| Bevington et al., 2020 | USA (National) | **End-users:**  Two rounds of focus groups (*n* = 95 and *n* = 73) and an online survey (*n* = 2050) were conducted with adult PA contemplators. | Qualitative Focus Groups (all participants) and a Quantitative Survey (only adults and parents) | **PA Guidelines (Children & Youth, Adults):**  The second edition of PA Guidelines for Americans recommending 60 minutes of MVPA per day, muscle-strengthening activities on $\geq$3 days per week and bone-strengthening activities $\geq$3 days per week for children & youth. It recommends 150-300 minutes of moderate-intensity or 75-150 minutes of vigorous-intensity PA or an equivalent combination per week and muscle-strengthening activities $\geq$2 days per week for adults. | The first round of focus groups assessed participants’ awareness and understanding of current PA guidelines, barriers and motivators to achieving them, and preference for information transfer. Findings informed the online survey.  The online survey assessed quantitative support of the focus groups and included questions regarding demographics, PA behaviours, goals, barriers, facilitators, and preferences for information transfer.  The second round of focus groups assessed participants’ responses to prototype messaging and imagery based on themes from the initial focus groups and survey.  The third round of focus groups assessed the translation of the preferred messages informed by the first two rounds of focus groups to additional audiences (age 3-16 years).  Focus group responses were analyzed for common themes. Findings from all 4 research activities were summarized together. | **PA Guidelines (Children & Youth, Adults):**  Adults, parents, teens, and children preferred messages that reflected diverse examples of activities; most participants disliked “one-size- fits-all” recommendations and would like to see the recommendation of 150min per week broken down into smaller goals. Adults and parents preferred messages that emphasized short- and long-term health benefits and preferred specific health benefits (treatment or prevention of specific diseases in short- or long-term) rather than generic messages about overall health. | RWR; 8/12 | |
| Birken et al., 2015 | Canada (Regional) | **End-users:**  *n* = 14 interviews of parents (3 fathers and 11 mothers) of children aged 1-5 years. | Qualitative Interviews | **SB Guidelines (Early Years):**  The Canadian Society of Exercise Physiology SB guidelines which recommends to limit prolonged sitting or being restrained (e.g., stroller, highchair) to <1 hour at a time in children 0-4. | Interview questions assessed stroller use, barriers and facilitators to stroller use, and participants’ perceptions of the relationship between stroller use and PA and health.  Interviews were audio-recorded, transcribed, and analyzed using thematic analysis. Themes and quotes were provided. | **SB Guidelines (Early Years):**  Parents reported that they use strollers as a means of transportation, storage of items, outdoor leisurely time, safe supervision, obtaining their own PA, and for a sleep setting away from home. Parent responses lacked consensus on the relationship between stroller use and PA. Many agreed with the guidelines that strollers reduced PA in children however some felt that it increased parent PA as well as promoted PA in children by using it a transportation device to play environments like the park.  Most parents didn’t think stroller use affected their children’s PA levels and were still able to ensure they were active outside of the stroller. | R | |
| Brown & Smolenaers, 2018 | Australia (National) | **End-users:**  *n* = 9 families were investigated consisting of 9 mothers and 2 fathers with child(ren) 2 and under with access to at least 1 screen. | Qualitative Interviews and a brief Quantitative Survey | **ST Guidelines (Early Years):**  Australian Government’s Move and Play Every Day: National PA Recommendations for Children 0-5 recommend that children <2 shouldn’t have any ST (television or other electronic media). | Interviews were conducted with each family to assess demographics, the number and types of screens in the house, and the amount of ST their child engaged in. Participants were then asked, “What are your thoughts on recommendation [to avoid ST at this age]?” Following the interview, they were provided with short surveys with questions that complemented the interviews.  Interviews were recorded, transcribed, and analyzed using a thematic coding framework. Themes and quotes were provided. | **ST Guidelines (Early Years):**  One third of parents were aware that a ST recommendation for children under 2 existed. Parents held a number of positive ideas about limiting their child’s time with television and other electronic media, yet also faced a number of challenges in implementing the recommendations. They felt that the recommendations were impractical and unrealistic. Parents also reported that ST at this age was difficult to quantify (irregular intervals of numerous screens across multiple environments). They weren’t sure what “counted” as ST, such as background television or the parent using the smartphone. Many parents also thought touch screens were developmentally beneficial. | R | |
| Carson et al., 2013 | Canada (National) | **Stakeholders:**  *n* = 331 practicing pediatricians | Quantitative Survey | **PA and SB Guidelines (Early Years, Children & Youth):**  2011 Canadian PA guidelines for early years (<1 year and 1-4 years) recommend at least 180 min of PA at any intensity spread throughout the day, including a variety of activities in different environments, activities that develop movement skills, and progression toward at least 60 minutes of energetic play by age 5. Children and youth (5-17 years) are recommended 60 minutes MVPA per day, vigorous-intensity PA $\geq$3 days per week and muscle- and bone-strengthening activities $\geq$3 days per week. The 2011 SB guidelines for the early years recommend to minimize the time infants (<1 year), toddlers (1–2 years), and preschoolers (3–4 years) spend being sedentary during waking hours. This includes prolonged sitting or being restrained (e.g., stroller, highchair) for more$\geq$1 hour at a time. For those <2 years, ST (e.g., TV, computer, electronic games) is not recommended. For children 2–4 years, ST should be limited to under <1 hour per day; less is better. Children aged 5-11 should minimize SB and limit recreational ST to <2 hours per day. | The survey included questions regarding how realistic the recommendations were for each age group and behaviour (‘very realistic’ to ‘unrealistic’), how feasible it would be to review the guidelines in a well-child visit (‘very feasible to not at all feasible’), and about the presence of any barriers to recommending the guideline to parents and caregivers during well-child visits. | **PA and SB Guidelines (Early Years, Children & Youth):**  Between 58% and 64% of the participants reported strong agreement with the PA guidelines and 69% with the SB guidelines. A range from 26% to 41% also agreed with the recommendations which means that over 90% of the sample either agreed or strongly agreed with the guidelines. X^2^ test revealed slighter more females (99%) agreed or strongly agreed with the early year guidelines compared to males (96%). Only 35% to 57% reported that the recommendations were very realistic for the guidelines. Of the pediatricians who perform well-child visits, 39% and 46% reported it would be very feasible to explain the guidelines for the early years and children respectively. The two most common barriers to recommending the guidelines to parents and caregivers were insufficient support or motivation from parents, caregivers, or youth (37%), and lack of time (24%). | 8/13 | |
| Carson et al., 2014 | Canada (Regional) | **End-users:**  *n* = 27 parents of children 4 years or under who attends a childcare centre. | Qualitative Focus Groups | **SB Guidelines (Early Years):**  2011 Canadian SB Guidelines for the Early Years (0-4 years) recommend to minimize the time infants (<1 year), toddlers (1–2 years), and preschoolers (3–4 years) spend being sedentary during waking hours. This includes prolonged sitting or being restrained (e.g., stroller, highchair) for $\geq$1 hour at a time. For those <2 years, ST (e.g., TV, computer, electronic games) is not recommended. | Participants were provided the formal definition of SB and given the Canadian SB Guidelines for the Early Years (age 0-4). The following interview guide was used to assess participants perceptions of the guidelines:  “1) What does SB mean to you? Is this definition similar/different than what you thought it was? 2) How clear are these guidelines? 3) What is your initial reaction to the guidelines? How do they make you feel as a parent (e.g., Irritated/ Frustrated/Happy/Guilty)? 4) Why do you think these guidelines are feasible/ unfeasible or realistic/unrealistic? 5) The guideline sheet lists several health benefits for young children associated with meeting the guidelines. What are your thoughts about these statements? Do you agree or believe them? 6) From what source would you need this information from to consider it credible/trustworthy? 7) How confident are you that your child could meet these guidelines? What barriers do you see? 8) How would you put the guidelines into practice? Are the suggestions on the bottom of the guideline sheet helpful? 9) How could the guidelines best be communicated/ presented to parents? 10) How could these guidelines be presented so that they were most helpful to you?”  Focus groups were audio recorded, transcribed, and analysed using a thematic analysis from Stewart et al. (2007). Themes and quotes were provided. | **SB Guidelines (Early Years):**  Most parents thought the guidelines were clear, comprehensive, and helpful. They supported the guidelines however, there was some confusion around the value of some sedentary activities for children such as reading and colouring as they aid in social and cognitive development. Parents also felt that there were several barriers in meeting the daily recommendations, such as demands of family life, screen technology and the environment where they live (e.g., weather). | R | |
| Evans et al., 2011 | USA (National) | **End-users:**  *n* = 180 children (6-13 years) and one of their parents | Qualitative Interviews | **ST Guidelines (Children & Youth):**  American Academy of Pediatrics recommendation of limiting children $\geq2$ television viewing to <2 hours a day. | Interviews were conducted in groups with parents of similar-aged children. Children of age 9-13 years participated in group interviews and those 6-7 years participated in one-on-one interviews.  During the interviews, researchers explored the reactions to the idea of instituting a 2-hour-a-day limited on television time.  Interviews were audio-taped, transcribed verbatim, and were analyzed using an iterative classification strategy and qualitative content analysis | **ST Guidelines (Early Years):**  Many participants had positive responses to the recommendation however they also had several concerns. The three themes of concerns were 1) the children’s negative reactions, including potential conflict between parents, children, and siblings, 2) a lack of parental resources, such as time, energy, money, and transportation, and 3) benefits to parents that are association with television viewing, including safe occupation while parents do household chores, a source of communication between parents and children, and regulation of the children’s behaviour. | R | |
| Faught et al., 2020 | Canada (National) | **Stakeholders:**  *n* = 877 guideline stakeholders (professionals involved with PA, SB, or sleep behaviour; e.g. policymaker, healthcare provider, public health practitioner, researcher, educator, recreation/sport practitioner) | Quantitative/Qualitative Surveys | **PA and SB Guidelines (Adults, Older Adults):**  A draft of the Canadian 24-Hour Movement Guidelines for adults aged 18-64 years and adults aged 65 years and older. Adults and older adults should both accumulate $\geq$150 minutes of MVPA per week, several hours of light PA per day, muscle-strengthening activities $\geq$2 days per week. Older adults should also do daily PA that challenges balance. Adults and older adults should limit SB to <8 hours, including <3 hours recreational ST and breaking up prolonged sitting. | Stakeholders were asked to fill in a survey that collected feedback on the guidelines. Participants were provided with the guidelines and then were asked to provide comments on each section. Key areas of feedback included importance, relevance, usefulness, advantages/disadvantages of the integrated approach, costs, benefits, and applicability to Canadians.  Qualitative responses were analyzed using inductive content analysis. | **PA and SB Guidelines (Adults, Older Adults):**  The majority of stakeholders thought that the guidelines were clearly stated. Many were concerned that the title and preamble had too high a literacy level, were too long, “movement” wasn’t a good descriptor of the behaviour, and more clarification was needed regarding the target audience. Stakeholders had apprehension that the PA guidelines were in a weekly format while the other health recommendations were in a 24-hour format and there was confusion of the statement “choose activities that challenge balance”. Regarding the SB recommendations, stakeholders wanted definitions and examples of “prolonged sitting” and “sedentary” and some thought this recommendation was unrealistic and/or intimidating. Stakeholders wanted a definition of “good quality sleep”, many thought that the sleep recommendation wasn’t realistic, and some were confused about the difference between recommendations for adults and older adults. Stakeholders reported that although guidelines were important, they were too challenging, impractical, and not useful in daily practice. They wanted more lay language and examples of varying levels of activities. Some stakeholders reported that the guidelines were not applicable to more complex populations, particularly those with varying socioeconomic status, accessibility, and ability. Some stakeholders commented that the integrated guidelines were overwhelming or confusing. | 4/14 | |
| Faulkner et al., 2016 | Canada (National) | **End-users, Stakeholders:**  *n* = 104 end-users and stakeholders (parents, teachers, pediatricians, qualified exercise professionals and youth between the ages of 5-17 years) | Qualitative Interviews, and Focus Groups | **PA and SB Guidelines (Children & Youth):**  A draft of the Canadian 24-Hour Movement Guidelines for Children and Youth (aged 5-17 years) were compared to the previous versions of PA and SB guidelines for children and youth. The recommendations are the same but the 24-Hour Movement Guidelines combines the recommendations from the prior PA and SB/ST guidelines. The PA guidelines recommend children and youth achieve 60 minutes of MVPA per day and vigorous PA, muscle- and bone-strengthening activities $\geq$3 days per week. The SB guidelines recommend children and youth should minimize SB each day and limit recreational ST to <2 hours per day. | Stakeholders were provided with the 2011 Canadian SB and PA Guidelines for Children and Youth (aged 5-17) and then were aske open-ended questions regarding the need of integrated guidelines (e.g., “What are your thoughts on a guideline that includes PA, sleep, and SB?”; “Would you find these integrated guidelines helpful or not helpful?”). Next, they were provided with the first draft of the Movement Guidelines and asked about the wording, their reactions, barriers to using them, and perspectives on dissemination (e.g., “Are there any barriers to implementing these guidelines at home or work?” “Who would be the best individuals to provide information about the guidelines to you?”; “What is the best way to present or communicate the guidelines?”).  Focus groups and interviews were audio recorded, transcribed, and analyzed using inductive data analysis. Themes and quotes were provided. | **PA and SB Guidelines (Children & Youth):**  The majority of participant responses indicated support of the Movement Guidelines. They thought the integration of behaviour provided a holistic view and appreciated the spectrum of levels of intensities of PA throughout the day. A few participants did not think it was necessary, particularly the youth participants who lacked interest.  Stakeholders identified a range of barriers to the uptake of the guidelines including concerns with accurately defining key terms such as "recreational" ST and various intensities of PA. The majority of parents reported stress and guilt surrounding the new guidelines related to the challenge of quantifying their children’s activities across various environments (school, day care, community centres). Stakeholders thought that the guidelines may be overwhelming to families and health practitioners and care would be needed to ensure the guidelines were communicated in a supportive and inclusive way rather than prescriptive. | R | |
| Gardner et al., 2017 | UK (National) | **End-users:**  n = 493 comments (unknown number of participants) | Comments from posts on UK news media websites | **SB Guidelines (Adults):**  UK guidelines for office workers to accumulate 2-4 hours of standing and light activity daily and to take regular breaks from prolonged sitting. | Five UK news media websites released reports on the SB guidelines for office workers on June 2, 2015. Comments from these posts were collected on July 1, 2015 and analyzed using narrative analysis. Comments were excluded if they were exclusive to the images that accompanied the reports. | **SB Guidelines (Adults):**  One of the three themes was “Challenges to the credibility of the sedentary workplace guidance” The comments challenged the strength of the evidence use to support the guidance, the credibility of its authors, and the applicability of the guidance to the real world. | R | |
| Golden et al., 2020 | USA (Regional) | **End-users:**  *n* = 12 (parents aged 18-40 with infants <12 months old, who use a smartphone daily | Qualitative Interviews | **ST Guidelines (Early Years):**  American Academy of Pediatrics ST recommendations to avoid ST for children <18 months and to limit use to video-chatting with an interactive adult. | One of the semi-structured interview questions was “How do you make sense of all the advice and counseling that you may receive regarding the upbringing of an infant?”  Interviews were recorded, transcribed and analyzed with thematic analysis. | **ST Guidelines (Early Years):**  When participants were alerted of the AAP ST recommendations, they reported little concern regarding the use of a smartphone in the presence of an infant. They reported it was inevitable that their child would engage with a smartphone since everyone, including the parents themselves as well as family and friends, has a phone on them all of the time. | R | |
| Hale et al., 2019 | Canada (Regional) | **End-users:**  *n* = 33 parents (31 mothers) of children <2 years | Qualitative interviews and a focus group | **ST Guidelines (Early Years):**  Canadian ST guidelines for children <2 years recommend no ST. | One component of the first objective of the interviews was to explore participants’ perspectives about infant obesity prevention education with a focus on the message of “no ST for kids under two”  Interviews were recorded, transcribed, and analyzed using thematic analysis.  Focus groups included some of the interview participants to ensure their views had been adequately represented. No new information was revealed by the focus groups. | **ST Guidelines (Early Years):**  Parents reported that although there are benefits to avoiding excessive ST, it was not realistic to avoid it completely. Further, ST is often used as a convenient distraction. Avoiding ST would mean staying away from many settings such as restaurants and other people’s homes. Many parents felt guilty about not allowing their child ST if they wanted it. Avoiding screens may be especially difficult for single or working parents, varying seasons, long car trips, and being in the presence of their older siblings. Parents preferred that the guidelines would allow for moderation. Some parents thought that the recommendations were too simple and wanted to know why the recommendations were the way they were. Parents feared that their children might miss out on the potential benefits of technology (e.g., educational programs). Simple messages with minimal text that were attractive with colourful graphics and provided more information for those interested was preferred. | R | |
| Handler et al., 2019 | Canada (National) | **End-users:**  *n* = 15 mothers of children with physical and/or intellectual disabilities (age 6-17 years) | Qualitative Interviews | **PA and SB Guidelines (Children & Youth):**  The Canadian 24-Hour Movement Guidelines for Children and Youth (5-17) recommend 60 minutes of MVPA per day and vigorous PA, muscle- and bone-strengthening activities $\geq$3 days per week. The SB guidelines recommend to minimize SB each day and limit recreational ST to <2 hours per day. | To explore how parents of children and youth with disabilities perceive the guidelines and explore whether parents consider the guidelines branding to be inclusive. Interview questions targeted language, images, information, and inclusivity of the guidelines.  Interviews were audio recorded, transcribed, and analyzed using deductive and inductive thematic analysis. Themes and quotes were provided. | **PA and SB Guidelines (Children & Youth):**  Most participants thought the concept of guidelines is valuable however they thought these guidelines are not inclusive or compatible with the abilities and needs of children and youth with disabilities.  They preferred the integrated guidelines and found them simple to understand.  However, they reported that the guidelines were difficult to implement among their children and youth with disabilities and there were discrepancies between what constitutes a certain level of intensity of PA. Participants wanted the guidelines to acknowledge differences in ability amongst the children and youth with disabilities.  They wanted elaboration of information regarding moderate-to-vigorous activity and light PA and examples of activities that meet these guidelines. They wanted information regarding accommodations and modifications for various activities in the guidelines. They suggested the use of an asterix to guide parents to additional resources and information as well as the addition of a phrase that acknowledges interindividual differences and that the guidelines may need to be modified to accommodate for this. Parents wanted the phrases “Sweat, Step” and “limited sitting for extended periods” to be replaced with other terms or phrases. | R | |
| Hattersley et al., 2009 | Australia (Regional) | **End-users:**  *n* = 63 adolescents (13-16years) and unrelated parents or primary caregivers of adolescents from low and middle socio-economic areas | Focus groups (9) | **ST Guidelines (Children & Youth):**  Australian ST guidelines recommend that children’s television viewing should be limited to 1-2 hours per day. | During the focus groups, participants were asked about their awareness and perception of the ST guidelines.  Focus groups were audio-recorded, transcribed verbatim, and analyzed with thematic analysis. | **ST Guidelines (Children & Youth):**  Most parents thought that the ST guideline wasn’t feasible, was ‘out of touch’ and wasn’t acceptable for adolescents. They questioned and criticized the basis of the guideline and one group dismissed health guidelines in general. Some parents were concerned of the achievability of the guideline. Adolescents thought that the guideline was unrealistic and unacceptable. | R | |
| Hinkley & McCann, 2018 | Australia (National) | **End-users:**  *n* = 28 parents of children 3-5 years | Qualitative Interviews | **PA and ST Guidelines (Early Years):**  Australian PA guidelines recommend interactive floor-based play and $\geq$ 30 minutes of tummy time per day for babies 0-12-months, $\geq$180 minutes of energetic play per day for children 1-2 years and $\geq$3 hours per day, with 1 hour being energetic play for children 3-5 years. ST guidelines recommend  children <2 years should avoid ST and children 3-5 years should keep ST to <1 hour per day. | During the interviews, participants were asked the following questions about ST or PA recommendations for young children:  “Can you tell me about the ST or PA recommendations for young children?”  “How appropriate do you feel the recommendations are?”  “Does ‘meeting the guidelines’ matter to you? Why/not? What does matter” | **PA and ST Guidelines (Early Years):**  Over half (60%) agreed that the guidelines for ST and PA were important. However, they also believe that individual practices within their family or their own childhood experience were what mattered most. | R | |
| Huxtable et al., 2018 | Australia (Regional) | **End-users:**  *n* = 15 parents of children 1-5 years from area of low socio-economic status and *n* = 2 nurses of the Maternal Child Health (MCH) program. | Case-study design with qualitative interviews | **PA and ST Guidelines (Early Years):**  Victorian State Government Maternal Child Health (MCH) 2009 Key Ages and Stages Framework. It includes recommendations for PA to encourage daily active play and to avoid ST for children <2 years. | During the interviews, parents were asked to describe their ease or difficulty in following MCH active play recommendations.  Interviews were audio-recorded, transcribed verbatim, and coded using a priori coding scheme. | **PA and ST Guidelines (Early Years):**  Nurses and parents reported that their children were already physically active and didn’t need further support. However, the recommendation of ‘no ST for 0-2-year old’s’ was difficult to follow. This was because screens were a part of adult life. Some parents stated they were strongly against large amounts of ST while others felt that they didn’t have strategies to prevent ST from their children. | R | |
| Irwin et al., 2005 | Canada (National) | **End-users:**  *n* = 71 parents of pre-school aged children (age 2.5-5 years) | Qualitative Focus Groups | **PA Guidelines (Early Years):**  2002 Canada's PA guidelines for pre-school aged children recommending 30-90 minutes of PA per day. Combine 3 types of PA (endurance, flexibility, and strength). | The focus groups assessed participants’ perspectives of their children’s PA behaviours  Focus groups were audio-recorded, transcribed, and analyzed using inductive content analysis. Themes and quotes were provided. | **PA Guidelines (Early Years):**  Parents perceived Canada’s PA Guidelines for Children as inadequate and that 30-90 minutes of PA per day was not enough. | R | |
| Jarvis et al., 2021 | Canada (National) | **End-users:**  *n* = 499 parents (377 mothers and 122 fathers) of at least one child 5-11 years | Quantitative survey | **PA and SB Guidelines (Children & Youth):**  Fact sheets of the Canadian 24-Hour Movement Guidelines for Children and Youth (5-17) recommend 60 minutes of MVPA per day and vigorous PA, muscle- and bone-strengthening activities $\geq$3 days per week. The SB guidelines recommend to minimize SB each day and limit recreational ST to <2 hours per day. | Participants were provided with fact sheets prepared by the Canadian Society of Exercise Physiology of the Canadian PA guidelines for children and a modified version of the Canadian ST guidelines for children (to contain only SB and ST behaviours). They then completed an online survey that measured the social issue advertising believability model constructs and perceived behavioural control. Message believability and attention were identified as perceptions of the guidelines. | **PA and SB Guidelines (Children & Youth):**  Message believability was high for the PA guidelines (5.79(1.20)) as well as the ST guidelines (5.72(1.23)). Mothers found both the PA guidelines (d = 0.39, 95% CI = 0.17-0.61) and ST guidelines (d = 0.22, 95% CI = 0.002-0.44) messages significantly more believable than fathers (P<0.01) with small effect size. No other effects were significant.  Attention was high or the PA guidelines (5.41(1.31)) and the ST guidelines (5.45(1.39)). No effects were significant and effect sizes were negligible. | 8 out of 12 | |
| Learmonth et al., 2019 | USA (National) | **End-users:**  *n* = 18 persons with multiple sclerosis | Qualitative Survey and Interviews | **PA Guidelines (Clinical):**  PA guidelines for persons with multiple sclerosis recommend 30 minutes of aerobic exercise 2x per week and resistance training exercise 2x per week. | The interviews and survey assessed research experiences, satisfaction with the program, exercise prescription, program materials, and behavioural change interactions, and recommendations for improvement.  One of the questions on the telephone interview was "What do you think of the guidelines?” (i.e., 30 min of aerobic exercise 2x per week and resistance training exercise 2 x per week).  Interviews were audio recorded, transcribed verbatim, and analyzed using inductive-category and thematic analysis. | **PA Guidelines (Clinical):**  Participants thought that the guidelines were achievable and appropriate and did not suggest changes. They liked that the guidelines allowed for flexibility and variety. Some of the participants suggested more variety in resistance exercises for progression. | R; 6/13 | |
| Martin Ginis et al., 2018 | Canada (National) | **End-users, Stakeholders:**  *n* = 45 persons with spinal cord injury and *n* = 13 spinal cord injury clinicians | Quantitative Survey | **PA Guidelines (Clinical):**  PA guidelines for adults with spinal cord injury.  For cardiorespiratory fitness and muscle strength benefits: $\geq$20 minutes of MVPA 2x per week and 3 sets of strength exercises for each major functioning muscle group, at a moderate- to vigorous-intensity, 2x per week.  For cardiometabolic health benefits: $\geq$30 minutes of MVPA 3x per week. | Stakeholders were provided a questionnaire that included 12 questions to evaluate their perceptions of the guidelines. Answers were on a 7-point Likert scale with higher scores indicating more positive ratings. | **PA Guidelines (Clinical):**  Stakeholders mean item responses were above the scale midpoint (i.e., > 4) for all of the questions, indicating positive agreement with the guidelines. The one exception was confidence that people with tetraplegia could meet the cardiometabolic guidelines. Scores were higher for the fitness guideline on items of appropriateness, confidence in achieving, and utility of the guidelines. The cardiometabolic guideline trended towards higher scores in clarity of instructions. | R; 5/10 | |
| Neher et al., 2020 | Sweden (Regional) | **Stakeholders:**  *n* = 48 Cancer rehabilitation professionals (*n* = 32 physiotherapists, *n* = 15 occupational therapists, and = 1 rehabilitation assistant) | Qualitative Focus Groups | **PA Guidelines (Clinical):**  PA guidelines of the national (Sweden) medical care program on cancer rehabilitation recommend 150 minutes of MVPA or 75 minutes of vigorous PA per week for cancer survivors; ongoing infection is an absolute contraindication to exercise; adapt the training during ongoing chemotherapy treatment or equivalent, as the daily form is strongly affected by side effects; in case of high susceptibility to infection, the training should be adapted in consultation with a doctor; in case of a known osteoporosis and extremely poor recovery during treatment, the training must be adapted to the circumstances. | During the focus groups, participants were asked about their perceptions of cancer rehabilitation, consequences of cancer and cancer therapy, and the barriers and facilitators for cancer rehabilitation practice.  Focus groups were audio recorded, transcribed, and analyzed using qualitative content analysis. Themes and quotes were provided. | **PA Guidelines (Clinical):**  One physiotherapist was surprised that the guidelines for cancer patients is the same as for healthy patients and another stated that they had never understood how very important the guidelines are. Some were doubtful of the applicability of PA principles to cancer survivors. | R | |
| Nobles et al., 2020 | UK (Regional) | **End-users:**  *n* = 11 adults; *n* = 5 older adults; *n* = 17 young people; n = 15 Somali women | Qualitative Participatory Workshops  . | **PA Guidelines (Children, Adults, Older Adults):**  2019 UK PA guidelines for children and young people (5-18 years) recommend 60 minutes of MVPA per day with a variety of types and intensities to develop movement skills, muscular fitness, and bone strength; adults (19-64 years) should accumulate $\geq$150 minutes of moderate intensity or 75 minutes of vigorous intensity PA per week and muscle-strengthening activities $\geq$2 days per week; older adults should accumulate $\geq$150 minutes of moderate intensity or 75 minutes of vigorous intensity PA per week and PA aimed at improving or maintaining muscle strength, balance, and flexibility $\geq$2 days per week. | Participants were put into workshops to collect information about how the guidelines and messaging could be better communicated to under-served community groups.  Researchers observed and took field notes and the workshops were audio recorded and transcribed. Together, these data were analyzed using the framework matrix. Themes and quotes were provided. | **PA Guidelines (Children, Adults, Older Adults):**  All participants felt that guidelines should include progressive steps towards achieving guidelines. They would like to see examples of different intensities of PA as well as images of different types of people and background. The Somali women thought that guidelines should be more culturally sensitive and include exercise examples that would be more appropriate to them such as indoor exercise with only other females and a female instructor. All groups found the language of the guidelines inaccessible and that words like “aerobic”, “intensity”, and “sedentary” weren't understood by everyone. Some participants preferred invitational messages in the guidelines that was encouraging and humorous while the Somali women preferred instructive messages. In general, all groups liked the phrase "some is good, more is better". | R | |
| Park et al., 2015 | South Korea (National) | **Stakeholders:**  *n* = 167 oncologists | Quantitative Survey Study | **PA Guidelines (Clinical):**  Exercise guidelines for cancer patients. ACSM recommends $\geq$150 minutes of MVPA or 75 minutes of vigorous-intensity PA per week, 2-3 weekly muscle-strengthening sessions, and flexibility sessions of stretching major muscle groups and tendons on days that other exercises are performed. Individuals with chronic conditions such as cancer should be as physically active as their abilities and conditions allow with some PA being better than none. | Oncologists were provided with two questionnaires: 1) to measure their attitudes towards recommending exercise to cancer patients that was scored with a 7-point Likert scale, 2) to measure perceptions of the benefits of exercise and barriers to recommending exercise to patients. | **PA Guidelines (Clinical):**  Oncologists (21%) reported one of the main barriers to recommending exercise to cancer patients was that exercise guidelines for cancer patients were unclear. | 5/11 | |
| Riazi et al., 2017 | Canada (National) | **End-users, Stakeholders:**  *n* = 10 stakeholders (physicians, early childhood educators in administration, PA communicators, and researchers who work with children 0-4 years)  *n* = 92 end users (parents, early childhood educators, and early childhood educator trainees) | Qualitative Interviews and Focus Groups | **PA and SB Guidelines (Early Years):**  A draft of the Canadian 24-Hour Movement Guideline for the early years (0-4 years). Infants <1 should be physically active several times in a variety of ways, obtain >30 minutes of tummy time throughout each day, not be restrained for >1 hour at a time, and have no ST. Toddlers 1-2 years should achieve 180 minutes of PA throughout the day, not be restrained for >1 hour at a time, and should have <1 hour of ST per day. Preschoolers 3-4 should achieve 180 minutes of PA throughout the day with $\geq$60 minutes of energetic play, not be restrained for >1 hour at a time, and ST should be <1 hour per day. | First, telephone interviews were conducted on stakeholders. Participants were provided with a draft of the Movement Guidelines and were asked about their first impressions, challenges and barriers to implementation, and dissemination methods and messengers. Sample questions were “What are the best ways to communicate the new guidelines to your particular constituency? How is information shared within your professional network? What resources do you need as a [interviewee’s job title] In order to provide information about the guidelines to parents?)”  Second, focus groups were conducted on end users. Participants were asked about their awareness of the PA and SB guidelines for the early years and then provided with a draft of the Movement Guidelines. They were asked about their first impressions, the clarity, and whether the integrated guideline was helpful. They also provided feedback regarding wording of the guidelines, their compatibility with daily life and work environments, challenges and barriers to implementation, and dissemination methods and messengers. Sample questions were “Do you find these integrated guidelines helpful or not helpful? How practical do you think these guidelines are for your work? Are there any barriers to implementing these guidelines? What are the best ways to present or communicate these guidelines? Who would be the best individuals to provide information to you about the guidelines?”  Interviews and focus groups were audio-recorded and transcribed verbatim. They were analyzed using thematic analysis. Themes and quotes were provided. | **PA and SB Guidelines (Early Years):**  Stakeholders and end users of all backgrounds supported the new guidelines and reported clarity and conciseness. Participants were in agreement with the divisions across age groups and the integration of the three movement behaviours (sleep, PA, and SB). They liked the recommendation to replace indoor time with outdoor time as well as the provision of specific goals to be met.  Although there was general receptivity toward the new guideline, many parents reported that the ST recommendation was difficult and perhaps unrealistic to achieve. They understood the negative effects of ST but explained that often used ST as a reward as a result of the child completing a certain task around the house. ECE's reported minimal difficulty in enforcing ST recommendations.  Many participants reported that the guidelines could cause guilt amongst the parents who find they don't have enough time amongst all of their other responsibilities to promote the guidelines amongst their children. That being said, most parents understood that the guidelines were more for striving towards rather than achieving every single day. Some parents needed more clarity around the fact that the 180 min of PA could be divided throughout the day. | R | |
| Sebastiao et al., 2015 | USA (Regional) | **End-users:**  *n* = 10 older African American Women (*n* = 5 active; *n* = 5 inactive) age 60-80 years | Focus groups and Simple Quantitative Survey | **PA Guidelines (Older Adults):**  PA guidelines for older adults retrieved from the Centers of Disease Control (CDC) and Prevention Website recommend 150 minutes of MVPA per week and $\geq$2 days of muscle-strengthening activities per week. If they cannot achieve this due to a chronic condition, they should be as physically active as their condition allows. The Exercise and PA: Your Everyday Guide from the National Institute on Aging (NIA) includes seven chapters that describe 1) the benefits and types of exercise, 2) goal setting, 3) how to stick to an exercise plan, 4) examples of exercises with instructions, 5) monitoring progress, 6) nutritious eating, and 7) additional resources. The Be Active Your Way: A Guide for Adults from the US Department of Health and Human Services includes three parts that describe personalized PA behavioural strategies for 1) inactive adults, 2) adults who aren’t as active, and 3) adults who are more active. | There were two focus groups, one for the active women (greater or equal to 150min MVPA per week) and one for the inactive women (less than 150min MVPA per week).  During the focus groups, participants were provided with 3 PA promotion brochures: 1) Exercise and PA: Your Everyday Guide from the NIA, 2) The CDC PA Guidelines for Older Adults, 3) Be Active Your Way: Your Everyday Guide from the US Department of Health and Human Services. Participants were asked to subjectively rate each brochure on a scale from 1 to 5 to assess clarity and content with 1 being poor and 5 being excellent. Following this, participants were asked about their perceptions and beliefs of the brochures using the following questions: “1) What are the brochures talking about?, 2) How did you feel after read these messages?, 3) Are the messages clear to you? Why or why not? 4) Did anyone feel the messages are somehow confusing? Difficult to understand? Why?, 5) Which of those do you relate to more? Why?, 6) Now think about older African American women in general. Would they be able to understand the message? Do you think your peers would have difficult in reading it/understanding it?, 7) What are some of the things we need to change, if any, in order to make the message clear to the African American community?, 8) What do you think about this document? What you ever seen it before? Does the information included motivate you? Does it catch your attention? What makes you interested in reading it or not interested in reading it [discussion of one document at a time].”  Focus groups were audio-recorded, transcribed verbatim, and analyzed using a descriptive thematic analysis based on realist and semantic methods. Themes and quotes were provided. | **PA Guidelines (Older Adults):**  There were no significant differences between active and inactive women regarding their perception of the clarity and content (scored from 1=poor to 5=excellent) of any of the brochures. The “Exercise and PA” brochure scored the highest with mean scores of 4.7(0.27) and 5(0) in the active and inactive groups respectively, the “Be Active Your Way Brochure” scored the second highest with mean scores of 3.4(0.42) and 3.8(0.83) in the active and inactive groups respectively, and the CDC PA guidelines scored the lowest with mean scores of 2.1(0.74) and 2(0.70) in the active and inactive groups respectively.  Participants in both groups found that the CDC PA guideline language was difficult to understand, the terminology was too technical, and they felt overloaded with information. In contrast to the CDC guidelines, the “Exercise and PA” and “Be Active Your Way” brochures were commended for including visual illustrations, providing examples, and having detailed explanations. Participants expressed concerns that the examples of activities across the brochures weren't reflective of African American people. | R; 7/11 | |
| Slater et al., 2010 | Australia (National) | **End-users:**  *n* = 1201 parents of children aged 2-16 years. | Interview that follows a 37-item survey with 24 closed and 13 open questions. | **PA and SB Guidelines (Children & Youth):**  Australian PA and SB guidelines for children recommended 60 minutes of PA per day and <2 hours of recreational ST per day. | Parents whether they thought it was realistic for their child to have less than 2 h of recreational ST on most days and could be physically active for at least 1 hr per day. They were asked to specify factors that might make it difficult to achieve the recommendations. | **PA and SB Guidelines (Children & Youth):**  Nearly all parents (92%) thought that the PA guidelines were realistic. Common barriers to meeting them included lack of time (38%), weather (29%), and lack of motivation or resistance from the child (7%).  Fewer parents (75%) thought the ST guidelines were realistic. Common barriers to meeting them included weather (12%), keeping children occupied (11%), the child’s enjoyment of the ST activities (10%), conflict as a result of limiting the child’s ST (9%), and difficulty monitoring ST (8%). | RWR; 8/13 | |
| Stanley et al., 2020 | Australia (National) | **End-users, Stakeholders:**  *n* = 35 end-users and stakeholders (parents of children (0-5 years), early childhood educators, and health policy professionals) | Qualitative Focus Groups and Key Informant Interviews | **PA and SB Guidelines (Early Years):**  Australian 24-Hour Movement Guideline for the early years (0-4 years). Infants <1 should be physically active several times in a variety of way, obtain >30 minutes of tummy time throughout each day, not be restrained for >1 hour at a time, and have no ST. Toddlers 1-2 years should achieve 180 minutes of PA throughout the day, not be restrained for >1 hour at a time, and should have <1 hour of ST per day. Preschoolers 3-4years should achieve 180 minutes of PA throughout the day with at least 60 minutes of energetic play, not be restrained for >1 hour at a time, and ST should be <1 hour. | Stakeholders were initially provided with the guideline to read through and familiarize themselves with it. Subsequently they participated in the focus groups and interviews where they were asked to discuss acceptability, perceived importance, clarity, implementation facilitators, implementation barriers, dissemination, and implementation strategies.  Focus groups and interviews were audio-recorded, transcribed verbatim, and analyzed using inductive thematic analysis. Themes and quotes were provided. | **PA and SB Guidelines (Early Years):**  Acceptability was one of the main themes identified amongst the focus groups. Stakeholders generally found the guidelines acceptable and were in favor of the integration of health behaviours and division across age groups (infants, toddlers, and preschoolers). There was discrepancy around time allocation of behaviours with educators and other professionals favoring its clarity while at the same time all of the stakeholders felt it may lead to negative feelings of guilt of the parents for not having their child meet the recommended times. Stakeholders reported that movement behaviour-related jargon such as "cognitive development" and "sleep hygiene" should be removed because it may be difficult for some people, especially those with low socio-economic backgrounds to understand this wording. Stakeholders suggested that the guidelines be presented in a visually appealing, inviting, and culturally appropriate way that emphasized artwork instead of text. | R | |
| Tennant et al., 2019 | Canada (National) | **End-users:**  *n* = 162 parents of children (5-17 years) | Prospective Randomized Experiment | **PA and SB Guidelines (Children & Youth):**  Canadian 24-Hour Movement Guidelines for Children and Youth (5-17 years) recommend 60 minutes of MVPA per day, vigorous PA and muscle and bone strengthening activities should each be incorporated $\geq$3 days per week, and they should have <2 hours of recreational ST per day, and limit sitting for extended periods. | Participants were randomly assigned to read one of three guidelines: 1) integrated guidelines, 2) segregated guidelines (4 guidelines for each of the behaviours), or 3) control guidelines (Transport Canada’s seatbelt guidelines for children and youth). They were asked to fill in pre-test and post-test surveys before and after reading the guideline as well as a two-week follow-up survey.  Participants indicated their perception of the guidelines as 1) enjoyable, 2) stressful, and 3) useful. A 7-point bipolar adjective scale was used (1 = extremely unenjoyable and 7 = extremely enjoyable).  Participants were asked to complete a Thought Listing task where they wrote up to 5 thoughts they had while reviewing the guidelines to assess message processing.  Recall, planning social cognitions (providing support to their child in meeting the PA guidelines), and PA behaviour of their children was also assessed.  Qualitative data was analyzed using thematic analysis. The percentage of themes reported by each participant was analyzed using univariate analyses of variance. | **PA and SB Guidelines (Children & Youth):**  There were no significant differences in perceptions, message processing, or recall of the guidelines between the groups (p>0.05). 2 of the 10 thought listing themes were “praise for the guidelines” and “criticism of the guidelines” and these thoughts were similar in frequency across the groups (p>0.05). | High Risk | |
| The Health Perspective, 2002 | USA (National) | **Stakeholders:**  *n* = 3  Dr. Steven N. Blair of the Cooper Institute in Dallas  Dr. Wotjtek Chodzo-Zajko, a former member of the World Health Organization's Scientific Advisory Committee  Colin Milner, CEO of the Vancouver-based International Council on Active Aging | Qualitative Interviews | **PA Guidelines (Adults):**  U.S. National Academy of Sciences/Institute of Medicine Guidelines for PA: At least 60 minutes each day of moderately intense PA such as walking/jogging at 4-5 mph. | Details of the interview protocol and questions were not provided.  Only quotes of responses were displayed. | **PA Guidelines (Adults):**  Dr. Blair thinks that is great that the Institute of Medicine included PA in its report on new dietary recommendations but he is concerned that the report could lead the public to not understand nor appreciate that relatively modest amounts of PA will improve health. He also states that people will obtain health benefits from 30 minutes of moderate intensity PA per day but they should build up to 60 minutes per day for added health benefits.  He worries that Americans won't still see the benefits in the previously recommended 3 times 10-minute walks per day. His understanding is that to increase the recommendation to 60 minutes is mainly for prevention of weight gain however this idea is not well supported by research.  Dr. Chodzko-Zajko stated that it isn't clear that 60 minutes of continuous PA is a necessary minimum threshold to meet for improvements in physiological, psychological, and social outcomes. In addition, 60 minutes per day may be unattainable by sedentary individuals. He thinks that blanket exercise recommendations don't do justice for a heterogenous population with varying levels of needs and abilities.  Colin Milner thinks that the guideline may increase barriers, resistance, and lack of understanding to exercise in the older adult population. | NR | |

*Note:* PA = physical activity, SB = sedentary behaviour, ST = screen-time

^1^Overall VAKS score: R = Recommended, RWR = Recommended with reservations, NR = Not Recommended

^2^Overall NIH Quality Assessment Tool for Observational Cohort and Cross-Sectional Studies (maximum score = 14)

^3^Overall RoB 2 score
