# Supplementary material for: Perceptions of physical activity and sedentary behaviour guidelines among end-users and stakeholders: a systematic review
Source: Int J Behav Nutr Phys Act. 2022 Mar 2;19:21. doi: 10.1186/s12966-022-01245-9 (PMC8889734; doi:10.1186/s12966-022-01245-9)
Supplement: Supplementary file 3 — Additional file 3: Supplementary Table 3. Codes for the Final Coding Scheme. [file 12966_2022_1245_MOESM3_ESM.docx]

**Supplementary Table 3**

*Codes for the Final Coding Scheme*

| Acceptance  Anger  Barriers  Believable  Building towards the guidelines  Children like screen time  Clarity  Confusion  Credibility  Definitions needed  Guilt  Integration of behaviours  Lay language  Layout  Mistrust  More specific (to developmental stage, ability)  Naturally active  Overall positive for physical activity guidelines  Overall positive for sedentary behaviour guidelines  Overall positive for screen-time guidelines  Overall negative  Positives associated with sedentary behaviours  Quantification and examples of behaviour needed  Reducing sedentary behaviour unrealistic  Simplicity  Socioeconomic status  Strength-based language  The why  Unable to control others’ screens  Unable to control workplace  Value |
| --- |
