# Supplementary material for: Perceptions of physical activity and sedentary behaviour guidelines among end-users and stakeholders: a systematic review
Source: Int J Behav Nutr Phys Act. 2022 Mar 2;19:21. doi: 10.1186/s12966-022-01245-9 (PMC8889734; doi:10.1186/s12966-022-01245-9)
Supplement: Supplementary file 4 — Additional file 4: Supplementary Table 4. Selected Quotes for Each Theme. [file 12966_2022_1245_MOESM4_ESM.docx]

**Supplementary Table 4**

*Selected Quotes for Each Theme*

| Guideline Target | Theme | Quotes |
| --- | --- | --- |
| Early Years | Overall positive support for PA and SB/ST guidelines. | *“It seems high but when you think about the amount of things that you do in a day then it is actually quite easy to reach that time. It does sound daunting, 3 hours is a long time. But when you break it down…”* (Parent) (Bentley et al., 2015)  *“[c]ertainly there are mornings, like this morning when I took him to daycare, I said to myself, ‘wouldn’t it be nice if I could just strap him in the stroller and get us there in like good time?’ But then he doesn’t get to… explore and walk and stuff like that as we go to daycare so I guess it’s not as fun for him being in the stroller, so we try not to use it for that reason.”* (Parent) (Birken et al., 2015)  *“It would be fine [to receive the recommendation]. Because if they have studied and know more than we do and have had experiences that [television] isn’t good, then yes. I would do everything in my power to avoid it because it’s for the good of my child.”* (Parent) (Beck et al., 2016)  *“It’s pretty obvious you shouldn’t be sitting your kids in front of the TV, especially with childhood obesity and everything”* (Parent) (Brown & Smolenaers, 2018) |
|  | Meeting the SB/ST guidelines is unrealistic. | *“When my husband’s working, I’ve got three kids and I’m trying to make supper, so [the television] is my babysitter…”* (Parent) (Carson et al., 2014) |
|  | Guidelines should be tailored to individuals and encourage achievable goals rather than rigid times. | *“Erm, reduced from what, though because I think that’s different for different personalities, because you’ve got some children, especially boys, you cannot get them to sit down and colour a picture because they’re just not interested but girls will quite happily kneel down and play with a dolls house for hours….I think I know it really is a blanket statement but I think in general boys and girls are different like that because of the you know the ﬁne motor skills for girls and then the large motor skills for boys”* (Parent) (Bentley et al., 2015)  *“I think it’s a good reminder – it’s goals, right? But you’re not going to reach this every day”* (Parent) (Riazi et al., 2017) |
|  | Guidelines should provide suggestions and visuals of examples that stakeholders can apply. | *“It depends on I guess what you say that it [PA] actually is. If it is just kind of playing. Yeah I think it’s very difﬁcult at that age to measure it… It does seem a lot though, but I would think, I have no idea how she compares to that”* (Parent) (Bentley et al., 2015)  *“My way of thinking with this is that if we were to put this in our QIP 13 [Quality Improvement Plan], and they come through and they say “Well, how are you 14 doing that?... we’ve got no documentation.”* (Educator) (Stanley et al., 2020)  *“…but to give them a pamphlet like that, honestly, I don’t even think it would get read; they’d just look at it and go, “Hm, whatever…”* (Parent) (Stanley et al., 2020) |
| Children & Youth | Conflicting positive and negative perception of the guidelines. | *“I would be real upset because that [ST guidelines] is not enough”* (Child) (Evans et al., 2011)  *“So you’re talking 14 hours [of television viewing] a week? Guess what? I think that’s realistic”* (Parent) (Evans et al., 2011)  *“…[the guideline] kind of makes you feel guilty. Honestly if my daughter watches a lot of TV, then this guideline is out of the window. She is also very active. When I read things like these [guidelines] I feel guilty that I am doing something that is detrimental.”* (Parent) (Faulkner et al., 2016) |
|  | End-users need more guidance on monitoring and integrating PA and SB guideline recommendations into their daily lives. | *“On the weekends [my child is] usually, you know, she’s by herself. She doesn’t have any other siblings so, I mean, unless I’m sitting down or, you know, have time to sit down with her she’s usually, you know, she uses that as a source of entertainment.”* (Mother) (Evans et al., 2011)  *“We knew what to do with them when they were younger. It’s hard to figure out what to do with them now. I mean, we can’t take them to the Franklin Institute [a science museum in the city] anymore—they’re bored with that.”* (Mother) (Evans et al., 2011)  *“I think if organized activities either through community centres or things like this, we’re able to state that this activity, one hour of gymnastics, accounts for MVPA of the guidelines. Parents would be like, “great I got it. My kid has got it taken care of.”* (Parent) (Faulkner et al., 2016)  *“I don’t know what light physical activity is. That’s the hard part where I go ‘does that include sitting at school and doing your homework or is that sedentary’?”* (Parent) (Faulkner et al., 2016) |
|  | Guidelines should be tailored to ability and developmental stage. | *“I guess limited sitting for extended periods is something that doesn’t ﬁt for a kid with disabilities.”* (Mother) (Handler et al., 2019)  *“I think the way it is set up now is perfect for kids without any disabilities, so like my typical child ....It just needs to go a little bit further for kids with disabilities.”* (Mother) (Handler et al., 2019)  *“It is unrealistic in its scope of what it is suggesting that children with disabilities can do”* (Mother) (Handler et al., 2019) |
| Adults | The PA and SB guidelines are too simplistic. | *“It looks like a coloring book. . . . The type of illustration and stuff, it just doesn’t feel right unless it is for kids”* (Adults) (Berry et al., 2010) |
|  | Guidelines need to be more understandable to varying literacy levels with focus on strength-based language. | *“No idea what ‘intensities’ are.”* (Adults’ workshop) (Nobles et al., 2020) |
| Older Adults | PA guideline content needs to apply more understandable and inclusive language. | *“Didn’t understand the word aerobic–associated with jumping around in a leotard.”* (Older adults’ workshop) (Nobles et al., 2020)  “*Words like moderate, vigorous, intensity, aerobic are scary/frightening.”* (Older adults’ workshop) (Nobles et al., 2020)  *“but some people might not know what moderate is, you know, to intensity… because you do have a lot of what you call the average Joe, they’re not middle class, they’re not gone to college, half of them really basically have not even finished high school… people like that, which is call the average Joe, you are going to have to do – break it down in just a little bit more, what they said, layman’s terms…”* (African American older woman) (Sebastiao et al., 2015) |
|  | Guidelines should be tailored to varying abilities and cultural backgrounds. | *“… the words, need to be brought down to another level, I think that some of it could be a little elevated for some people… if the seniors, they may be having problems with the reading… so they can’t relate or read all those words…some African American descendent would have trouble with this… it’s way too much information, it’s information overloaded…”* (African American Older Woman) (Sebastiao et al., 2015) |
| Clinical Populations | Guidelines are unclear for cancer survivors. | *“I wouldn’t really be able to say how physically active I think these patients should be compared to others…”* (Oncology Physical Therapist) (Neher et al., 2020) |
|  | Overall positive support for PA guidelines that target persons with multiple sclerosis (MS) and persons with spinal cord injury. | *“I think the exercise guidelines are appropriate. Not that one size fits everybody obviously, but I think that that's a very reasonable expectation, and I think it is something that can be accomplished by most people”* (Person with MS) (Learmonth et al., 2019) |

*Note:* PA = physical activity; SB = sedentary behaviour; ST = screen-time
